# Remote sensing and geochemical constraints on polymetallic mineralization in Abu Rusheid and Sikait granites of Egypt

**DOI:** 10.1038/s41598-026-40638-9

**Published:** 2026-02-27

**Authors:** Saif M. Abo Khashaba, N. H. El-Shibiny, Safaa M. Hassan, Kirsten Drüppel, Mokhles K. Azer

**Affiliations:** 1 Geology Department, Faculty of Science, Kafrelsheikh University, Kafrelsheikh, 33516 Egypt; 2 National Authority for Remote Sensing and Space Sciences (NARSS), Cairo, 1564 Egypt; 3 Institute of Applied Geosciences, Karlsruhe Institute of Technology, Adenauerring 20b, 76131 Karlsruhe, Germany; 4 Geological Sciences Department, National Research Centre, Cairo, 12622 Egypt

**Keywords:** Machine learning, Hyperspectral PRISMA, Rare metals, Columbite, ANS, Zinnwaldite, Environmental sciences, Solid Earth sciences

## Abstract

The post-tectonic granites and pegmatites of the Abu Rusheid-Sikait area, in the South Eastern Desert of Egypt, represent highly mineralized plutons within the Arabian Nubian Shield (ANS). This study integrates remote sensing datasets, field work, petrographic, and geochemical analyses to investigate these plutons. Machine learning algorithms (MLAs), including Support Vector Machine (SVM) and Random Forest (RF), applied to Minimum Noise Fraction (MNF)-enhanced PRISMA data, successfully discriminated lithological units with an overall accuracy of up to 89%. Spectral analysis identified four main hydrothermal alteration zones (phyllic, argillic, propylitic, and ferrugination), which were validated by field and laboratory data. Geochemically, the post-collisional granites show geochemical characteristics of highly evolved peraluminous A-type granites generated in a within-plate environment similar to other A-type granites in the ANS. All the studied granites display negative Eu/Eu* (0.02–0.19) anomalies, reflecting plagioclase fractionation. The geochemical similarities between the pegmatites and the surrounding granites support their genetic relation. Zinnwaldite-muscovite and garnet-muscovite granites, along with associated pegmatites, are highly enriched in rare earth and high field strength elements (e.g., ΣREEs up to 1310 ppm, Zr up to 4477 ppm, Nb up to 1500 ppm, Ta up to 216 ppm, U up to 411 ppm). This mineralization occurs as both disseminated accessory minerals within host granitoids and concentrated along structurally controlled zones that are affected by the Nugrus shear zone, faults (NW-SE, N-S, and NE-SW), and fractures, as confirmed from automatic surface structure lineament extraction maps, field work, and petrography. The enrichment is primarily controlled by extreme magmatic fractionation, which produced primary rare metal-bearing minerals (zircon, columbite, xenotime, monazite). This magmatic signature was subsequently overprinted by hydrothermal alteration, which redistributed and further concentrated metals along structurally controlled pathways concentrated metals along brittle fracture zones formed by the late-stage reactivation of the Najd Fault System, forming secondary minerals like kasolite and galena. Ferrugination is considered the main alteration related to uranium and REEs remobilization and concentration in specific locations, sometimes alongside iron oxides (hematite, goethite). This work presents an integrated model that links magmatic processes, structural controls, and hydrothermal alteration, providing a valuable framework for rare metal exploration in the ANS.

**Supplementary Information:**

The online version contains supplementary material available at 10.1038/s41598-026-40638-9.

## Introduction

Post-collisional A-type granites have gained significant attention due to their association with strategic metals essential for advanced technologies, including Zr, Nb, Ta, Li, Y, Th, U, and REEs^1–12^. In the Arabian-Nubian Shield (ANS), they formed during the end-stages of magmatic activity and cratonization in the Pan-African orogen (635 − 590 Ma)^2,13,14^. Some of these granites have been affected by post-magmatic hydrothermal alteration, which is often linked to rare metal mineralization. Among these, the Abu Rusheid-Sikait area is considered a promising site for exploration; however, critical knowledge gaps remain regarding the complex relationships between granite petrogenesis, structural controls, and alteration processes, as well as the enrichment of rare metals in these systems. Specifically, the spatial distribution of different granite plutons, the extent of hydrothermal alteration, and the structural pathways controlling mineralization are poorly constrained. The integration of advanced remote sensing technologies with traditional geological methods offers unique opportunities to delineate these complex relationships at multiple scales.

**Fig. 1:**
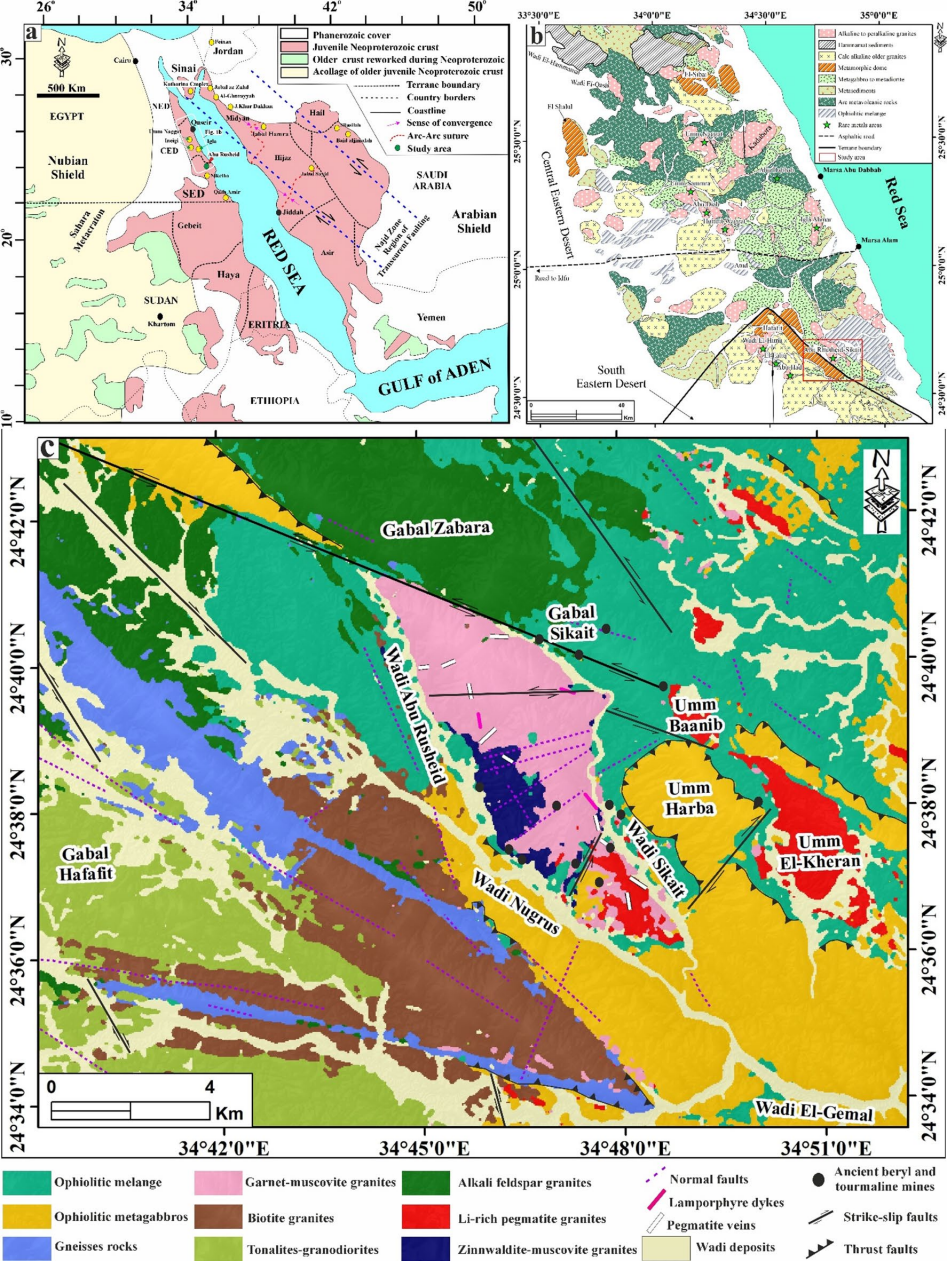
(a) Location map of the Arabian Nubian Shield (ANS) showing the distribution of rare metal granites as well as the location of the Abu Rusheid-Sikait area, modified after Johnson et al.^44^. (b) Geologic map of the Eastern Desert of Egypt, including the study area, modified after Abo Khashba^1^ and Stern and Ali^46^. (c) Geological map of the Abu Rusheid-Sikait area based on the present work (integrated machine learning algorithms (PRISMA-based SVM-MNF), field observation, petrography, geochemistry), and previous works^47,48^. The figure was created by ArcGIS Desktop 10.8. (https://www.esri.com/enus/arcgis/products/arcgis-desktop/overview/).

The Egyptian granites are widely diverse, and their compositional characteristics are influenced by several factors, including magma source, the degree of partial melting, and crustal contamination^1–6,8,9,15,16^. In Egypt, more than 18 rare metal-bearing granitoids and related pegmatites are known (Fig. 1a, b; Supplementary 1). Lithospheric delamination, asthenospheric upflow, and slab break-off processes are commonly assumed to characterize the post-collisional evolution of the ANS. Their emplacement has been structurally controlled by the NW-SE Najd fault system, together with conjugate NE-SW and E-W striking faults^4,5,8,9,13^.

Remote sensing techniques have become essential tools in modern geoscience, particularly for mineral exploration, lithological mapping, and identifying surface structural features^4,5,17–28^. The integration of hyperspectral PRISMA (PRecise Imaging Spectral Mapping for Applications) data, radar imagery (ALOS PALSAR, Sentinel-1), and field spectroscopy (ASD) provides a powerful framework for addressing fundamental questions about granite-hosted rare metal systems. This approach utilizes calibrated reflectance spectra captured across hundreds of narrow, contiguous wavelength bands spanning the VNIR to SWIR ranges, making it highly effective for mineral detection^29,30^. Accordingly, the processed remotely sensed datasets are used to detect several types of mineralization in metallogenic zones worldwide, like rare earth elements (REEs)^1,4,5,8–10^, lithium deposits 22, gold^24–27^, and porphyry copper deposits^23,28^.

Machine learning algorithms (MLAs), including Random Forest (RF) and Support Vector Machines (SVM), can be used effectively in accurate automatic lithological mapping using different remote sensing datasets like hyperspectral data (e.g., PRISMA) or multispectral data (e.g., Landsat OLI, Sentinel-2, and ASTER)^5,19–21,30–33^. The multispectral sensors are unable to distinguish between the wide variations in mineral compositions distinctly, as they have a limited number of bands, particularly within the SWIR range (2 to 6 bands in most cases). In contrast, hyperspectral sensors like PRISMA provide continuous spectral coverage between 400 and 2500 nm, allowing for the identification and discrimination of rock types based on their unique spectral signatures^19,20,34–38^. This significantly enhances lithological classification by capturing subtle mineralogical differences often overlooked by traditional methods. Furthermore, PRISMA data can be analyzed automatically using machine learning techniques, improving the accuracy and efficiency of geological mapping. The use of PRISMA for lithological discrimination not only enhances our understanding of geological formations but also facilitates the targeted exploration of mineral resources^19,20,38^.

This study introduces a comprehensive integration of high-resolution PRISMA-derived lithological and alteration maps with detailed fieldwork, petrographic analysis, and geochemical data. This integrative approach enables a quantitative investigation of the complex interplay between primary magmatic enrichment and subsequent structurally controlled hydrothermal overprinting in the Abu Rusheid–Sikait area.

In this study, we aim to achieve the following objectives: (1) accurately map the various lithological units utilizing machine learning algorithms applied to the hyperspectral PRISMA data, (2) delineate and characterize distinct alteration zones associated with rare metal-bearing granitoids using PRISMA spectral band indices and field spectroscopy (ASD) as well as surface structural lineaments based on ALOS PALSAR and Sentinel-1 radar datasets combined with petrographic investigations, (3) elucidate the magma sources and tectonic setting of the granitoids, and (4) evaluate the enrichment processes of the rare metal mineralization in the granitoids. We applied this metallogenic understanding to map new potential areas. Collectively, these efforts will provide a comprehensive understanding of the factors controlling rare metal mineralization in the Abu Rusheid-Sikait area.

## Geological setting

### Geodynamic framework

The East African Orogeny (EAO) is the main stage of the Neoproterozoic assembly of East and West Gondwana (Australia–India–Antarctica and Africa–South America), also including the Arabian Nubian Shield (ANS). Based on the plate tectonic theory, several models for the evolution of ANS have been proposed^39-44^. The ANS evolved through different tectono-magmatic stages^44,45^: (1) Basement and metasedimentary wall-rocks (ca. 900–870 Ma): deposition, metamorphism, and deformation of arc-derived turbidites and older supracrustal sequences (including greywackes, pelites, and minor carbonate layers) that would later be transformed into high-grade gneisses and migmatites. These metasedimentary protoliths record pre-ophiolite sedimentation along a passive-margin or back-arc basin margin and form the wall-rock assemblage against which ophiolitic slices were emplaced. (2) Oceanic ophiolites stage (ca. 870 − 750 Ma) that is characterized by a basal mantle section (peridotites and tectonized peridotites) overlain by mafic rocks of oceanic crust (gabbros, sheeted dykes, and mafic volcanic rocks); (3) Island-arc stage (ca. 770 − 650 Ma) represented by a series of mafic to intermediate rocks, including volcanic sequence and gabbro-diorite complexes, (4) syn-to late-orogenic stage (ca. 650 − 635 Ma), represented by emplacement of calc-alkaline plutonic rocks (granodiorites-tonalites) and volcanics of andesite to rhyolite compositions (Dokhan volcanics), and (5) post-orogenic stage [ca. 635 − 590 Ma; 13, 14] features two magmatic suites with considerable temporal overlap. The first, the primary focus of this work, consists of calc-alkaline to alkaline rocks and was emplaced between 635 and 590 Ma. The second, characterized by alkaline affinity, formed from 610 to 590 Ma. The intraplate magmatic activity continued during the Phanerozoic period, leading to the formation of small plutons, ring complexes, dykes, sills, plugs, and lava flows.

### Geology of the study area

The Abu Rusheid-Sikait area is located within the Neoproterozoic ANS of the Egyptian Eastern Desert (Fig. 1a) and forms part of a wider range of rare-metal-bearing granitic plutons, documented by Johnson et al.^44^. The area is delineated by latitude 24°33’45” N to 24°43’06” N and longitude 34°39’14” E to longitude 34°52’11” E, covering an area of ca. 385 km^2^ (Fig. 1), ca. 120 km southwest of the Marsa Alam City (Fig. 1b). Many authors^13,15,51–67^ have previously studied the geology, petrology, geochemistry, and structural setting of the study area. The study area is structurally constrained by the Sikait-Nugrus fault system, including the Nugrus thrust fault 15, 49, 60, 62, 67, also called the Nugrus strike-slip fault^50,67^, and/or the Shait-Nugrus shear zone^15,51,60^(Fig. 1b, c). Additionally, several thrusts and shear zones align along a NW–SE trend, resulting from the Najd Shear System^15,60,62,65–67^. Steep slopes and rugged mountains characterize the area, as well as a number of wadis, including Wadi Abu Rusheid, Wadi Sikait, and Wadi Nugrus (Fig. 1c). Exposed rocks comprise Neoproterozoic rocks, including gneisses, ophiolitic metagabbros, ophiolitic mélange suites, and syn- to post-collisional granitoids [685 to 629 Ma; 13, 54], which were later intruded by lamprophyre dykes, as well as pegmatite and quartz veins (Fig. 1c). Three prominent shear zones crosscut the gneissic rocks, with the first two being oriented in an NNW-SSE direction and the third in an ENE-WSW direction^55^. The gneissic rocks are known to host mineralization, including uranium and associated minerals^55,57^. Ophiolitic metagabbros were superimposed on the ophiolitic mélange in a WNW-ESE direction (Nugrus thrust fault) at low to high angles of up to 30°^59^.

**Fig. 2:**
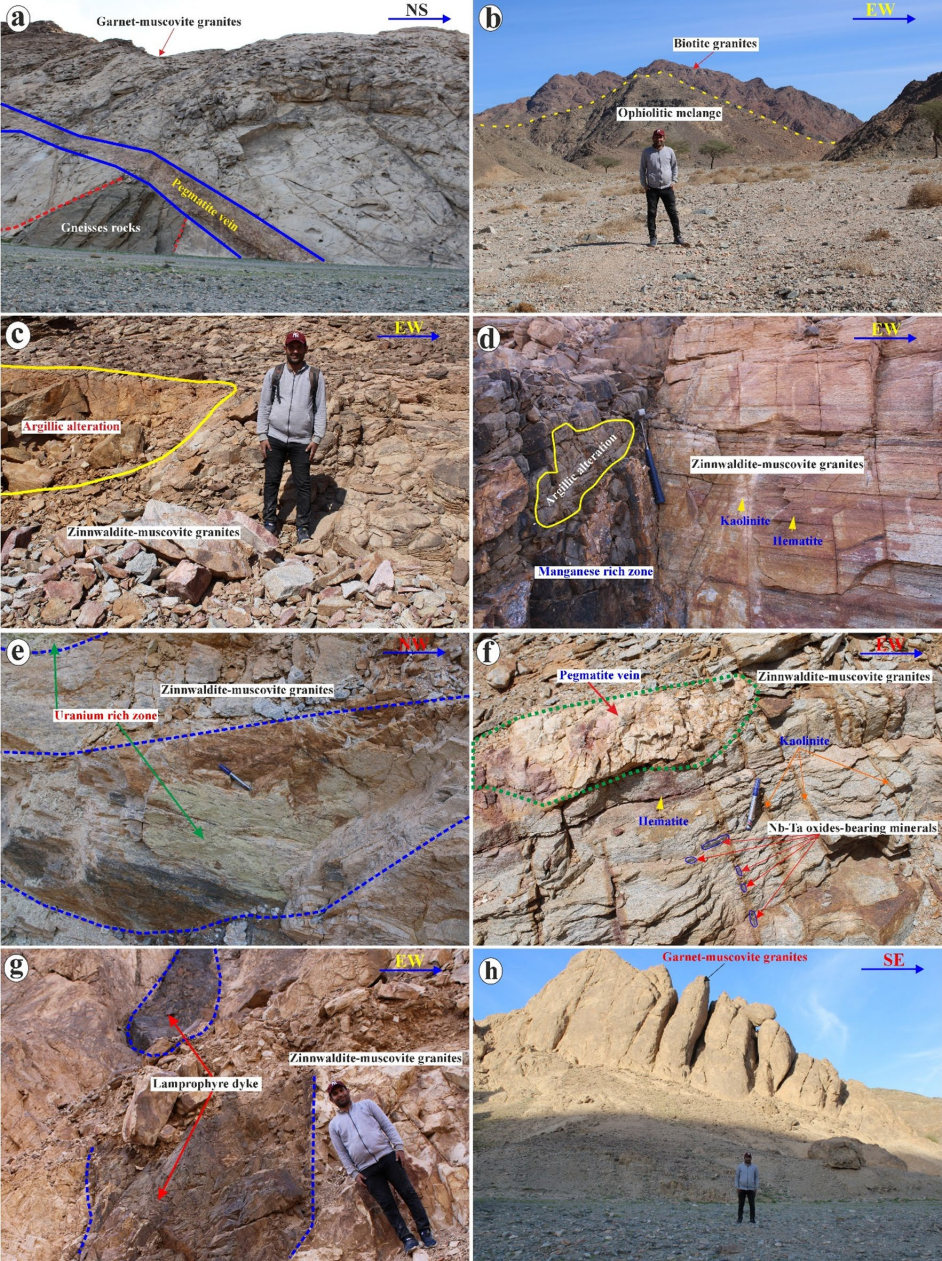
Field observation of the Abu Rusheid-Sikait area. (**a**) Panorama view of the intrusive contact between gneissic rocks and zinnwaldite-muscovite granites that cut by a large pegmatite vein. (**b**) Panorama view of the contact between biotite granites and ophiolitic mélange. (**c**) Large argillic alteration in zinnwaldite-muscovite granites. (**d**) Close-up view of the argillic alteration, manganese-rich zone, and hematization in zinnwaldite-muscovite granites. (**e**) Close-up view of fracture-filling yellowish-green uranium-rich zone in zinnwaldite-muscovite granites. (**f**) Clos-up view of pegmatite vein cutting zinnwaldite-muscovite granites with the occurrence of Nb-Ta oxide minerals at the fractures. (**g**) Lamprophyre dykes cut zinnwaldite-muscovite granites. (**h**) Garnet-muscovite granites of the Wadi Sikait. These photos are our own, and we agreed to publish them.

In this study, a detailed and accurate geological map of the Abu Rusheid-Sikait area (Fig. 1c) was produced using support vector machine (SVM) algorithms applied to the minimum noise fraction (MNF) of hyperspectral PRISMA data, “refer to Sect. 4.1.1” based on field investigation, petrography, geochemistry, and previous works^47,48^. The gneissic metamorphic rocks in the study area exhibit a pronounced ENE-WSW trending foliation and are thrust down the ophiolitic mélange with a well-defined tectonic contact (Fig. 1c). The gneisses cover the core region of an underlying granitic pluton (Fig. 2a). The ophiolitic mélange in the area is composed of ultramafic rocks and layered metagabbros surrounded by metasedimentary units and displaying sharp intrusive contacts with younger granitic rocks (Figs. 1c and 2b). Older syn-collisional granitoids [ca. 685–665 Ma; 56] are represented by tonalites-granodiorites (Fig. 1c). On the other side, the post-orogenic activity in the study area, which dates 629.3 ± 14 Ma using U-Pb zircon geochronology^13^, is marked by the emplacement of a different suite of igneous intrusions, including biotite granites, garnet-muscovite granites, zinnwaldite-muscovite granites, Li-rich pegmatite granites, and alkali feldspar granites (Fig. 1c). The area is further dissected by numerous pegmatite and quartz veins, as well as lamprophyre dykes (Fig. 1c).

**Fig. 3:**
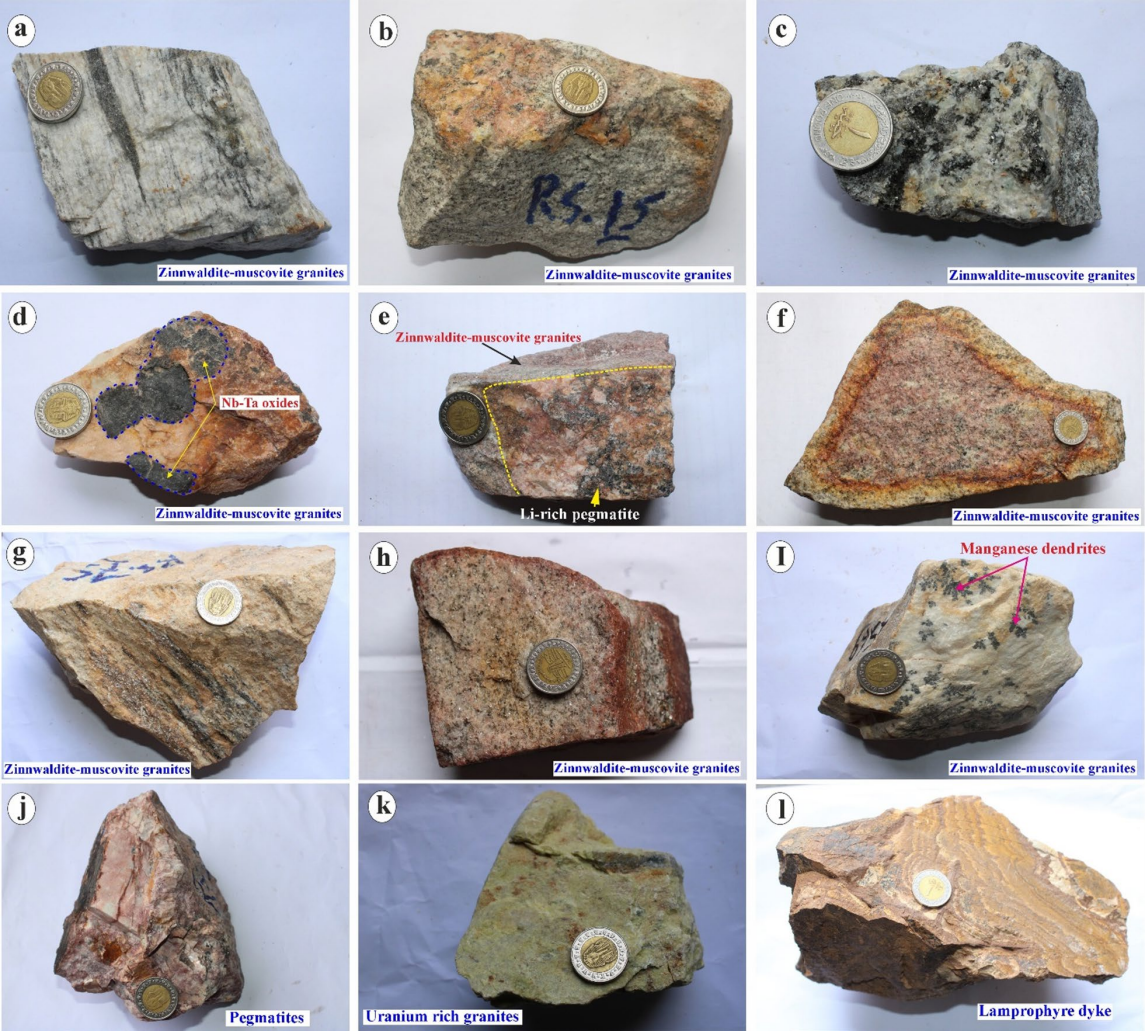
Hand samples of some interesting features in the studied granitoids. **a**) Zinnwaldite-muscovite granites with well-developed foliation defined by aligned mica flakes and quartz-feldspar matrix. **b**) Zinnwaldite-muscovite granites displaying patchy hematization and orange staining, indicative of ferruginous alteration. **c**) Zinnwaldite-muscovite granites containing abundant dark biotite and secondary iron oxides along grain boundaries. **d**) Zinnwaldite-muscovite granites with irregular clusters of Nb-Ta oxide minerals (columbite-group) highlighted by dashed outline. **e**) Contact between zinnwaldite-muscovite granites and a Li-rich pegmatite vein (yellow outline), showing sharp intrusive boundary. **f**) Strongly hematized zinnwaldite-muscovite granites with pervasive red-brown staining from iron oxide minerals. **g**) Zinnwaldite-muscovite granites cut by narrow quartz veinlets with minor sericite alteration along margins. **h**) Zinnwaldite-muscovite granites exhibiting mottled alteration halos of red and yellow iron oxide minerals along small fractures. **i**) Zinnwaldite-muscovite granites hosting dendritic manganese oxide precipitates (pink arrows) on weathered surfaces. **j**) Coarse-grained, pink-to-white pegmatite with blocky K-feldspar, quartz, and muscovite intergrowths typical of rare-metal pegmatites. **k**) Uranium-rich granite sample showing bright yellow secondary uranium mineral staining (kasolite-fluorite) on the surface. **l**) Fine-grained lamprophyre dyke cutting the granite, with penetrative flow foliation and dark mafic mineral matrix. These photos are our own, and we agreed to publish them.

These post-orogenic granites, the focus of this study, are characterized by a massive and homogeneous texture, varying from medium- to coarse-grained, and exhibit colors ranging from white, pinkish-white, grey, yellowish-grey, light grey to deep red (Figs. 2 and 3). Biotite granites occur mainly in the southwestern part of the mapped area, covering an area of ca. 42 km^2^, and display sharp intrusive contact with both the gneissic country rocks and the ophiolitic units (Fig. 2b), while they exhibit gradational contacts with the garnet-muscovite granites (Figs. 1c). Muscovite granites (ca. 22.5 km^2^), mainly exposed between Wadi Abu Rusheid and Wadi Sikait, display sharp intrusive contacts with the gneisses rocks, the ophiolitic metagabbros, and mélange, and a gradational contact with biotite granites (Figs. 1c and 2a and c). They are cross-cut by numerous normal faults and are frequently invaded by pegmatites, quartz, tourmaline veins, and lamprophyre dykes (Fig. 1c). Zinnwaldite-bearing granites (ca. 4.2 km^2^) are exclusively observed in the marginal, strongly sheared parts of the muscovite granites, especially in the Wadi Abu Rusheid and Wadi Sikait areas (Fig. 1c).

As a result of tectonic activity in the area, the marginal parts of many granite plutons are transected by shear zones (Fig. 1c). The studied post-collisional granites were affected by strong alteration, which is apparently mainly controlled by the NW-SE trending Najd fault system and conjugate NE-SW and N-S trending faults (Fig. 2d, e). In the field, the mineralized zones in granites and associated pegmatites are visible on the macroscopic scale, as indicated by color changes (Figs. 2e-g and 3a-i). Specific mineralization zones, such as a manganese-rich zone (Figs. 2d and 3i), a uranium-rich zone (Figs. 2e and 3k), and a rare metal-rich zone, are observed close to fractures (Figs. 2f and 3d).

Post-collisional granites are cross-cut by numerous pegmatites and quartz veins, as well as lamprophyre dykes (Figs. 1c and 2a, f and g). Pegmatite veins consist mainly of coarse-grained K-feldspar, quartz, and micas and are rich in rare metals-rich minerals (Figs. 2a, d and f and 3j). Pegmatites commonly occur as NNW-SSE and ENE-WSW trending veins, which range in length from ca. 50 cm to several meters (Fig. 2a, f). Moreover, they can be found as pockets or flat lenses (ca. 25 m in length) along the margins and in the core region of the garnet-muscovite granites, zinnwaldite-muscovite granites, and in the ophiolitic mélange (Fig. 2a, f). Li-rich pegmatites occur in three localities in the study area, including (1) the southern part of muscovite granites between Wadi Abu Rusheid and Wadi Sikait, (2) in the Wadi Umm El-Kheran pluton, and (3) in the Umm Baanib pluton, covering an area of ca. 10.8 km^2^ (Figs. 1c and 2h). They are characterized by coarse-pegmatitic grain sizes and are rich in zinnwaldite, muscovite, and garnet. They show gradational contacts with zinnwaldite-muscovite granites and sharp intrusive contacts with other rock units (Fig. 1c). Lamprophyre dykes are fine-grained, vary in thickness and colors (black, black-grey, whitish, and pink), and trend mainly in the NNW-SSE direction (Figs. 1c, 2g and 3l).

Ferrugination zones are particularly well-developed in zinnwaldite-muscovite granite exposures, forming discontinuous patches ranging from 2 to 15 m in diameter, often associated with uranium-rich secondary mineral concentrations visible as bright yellow surface staining over areas of 1–5 m² (Figs. 2d-f and 3). The Nb-Ta oxide mineralization occurs as millimeter- to centimeter-scale disseminated grains within pegmatite veins (0.5–3 m width, 10–200 m length) and as concentrated pods at fracture intersections, with the highest concentrations observed at the contact zones between zinnwaldite-muscovite granites and cross-cutting pegmatite bodies (Figs. 2a and f and 3). Moreover, the phyllic alteration is mainly concentrated in the muscovite-rich granites like garnet-muscovite granites and zinnwaldite-muscovite granites (Figs. 2a and c-h and 3).

## Materials and methods

### Remotely sensing data

This work utilized the hyperspectral PRISMA dataset for accurate lithological mapping and identifying alteration zones linked to rare metals mineralization in the Abu Rusheid-Sikait area in the northern ANS. The PRISMA hyperspectral data (PRS_L2D_STD_20200817083141_20200817083146_0001) used in this study was acquired on August 17, 2020, and downloaded from the mission website (www.prisma.asi.it). As described in the specifications for PRISMA (see www.prisma.asi.it), L2D PRISMA products are atmospherically corrected images based on the ASI standard data processing chain. In this investigation, hyperspectral PRISMA data was employed to automatically delineate lithological rock units utilizing machine learning algorithms. Using two radar datasets, ALOS PALSAR and Sentinel-1B, the structural features associated with alteration and mineralization in the Abu Rusheid-Sikait area were detected. In this study, we employed the Enhanced Lee filter to reduce speckles and enhance geological contacts in ALOS PALSAR and Sentinel-1 images. Moreover, surface lineaments were mapped using the LINE module in PCI-Geomatica software, applied to cross-polarized (HV) data from both datasets. Rose diagrams for both datasets were produced using RockWorks software, version 2018. Mineral spectral analysis of fourteen granitoid samples from the Abu Rusheid-Sikait area was conducted using the ASD TerraSpec Halo Mineral Identifier Spectrometer, a portable field device optimized for rapid mineral characterization.

### Fieldwork and petrography

According to pre-field remote sensing studies, a total of 140 samples were collected from different granitic varieties, pegmatites, alteration zones, lamprophyre dykes, and quartz veins. We prepared seventy thin- and polished sections to investigate the mineral assemblages, textures, and hydrothermal alteration types of the collected specimens, as well as selecting a subset for microprobe mineral analyses.

### Geochemistry

#### Mineral chemistry

A detailed electron microprobe analysis (EMPA) of the feldspar, mica, zircon, columbite, and topaz in the zinnwaldite-muscovite granites and biotite granites of the Abu Rusheid-Sikait area was conducted at the Department of Earth Sciences, University of Western Ontario, Canada. These analyses were conducted using a JEOL JXA-8530 F field-emission electron microprobe.

#### Whole-rock chemistry

Based on the petrographic study, a total of twenty-six representative samples from the Abu Rusheid-Sikait granites and pegmatites were selected for whole analyses, encompassing major, trace, and rare earth elements (REEs). The concentrations of major and trace elements were determined using a ThermoARL X-ray fluorescence (XRF) spectrometer, while the REEs and other trace elements were measured with an Agilent 7700 inductively coupled plasma mass spectrometer (ICP-MS). All analyses were performed at the GeoAnalytical Laboratory at Washington University, USA.

**Fig. 4:**
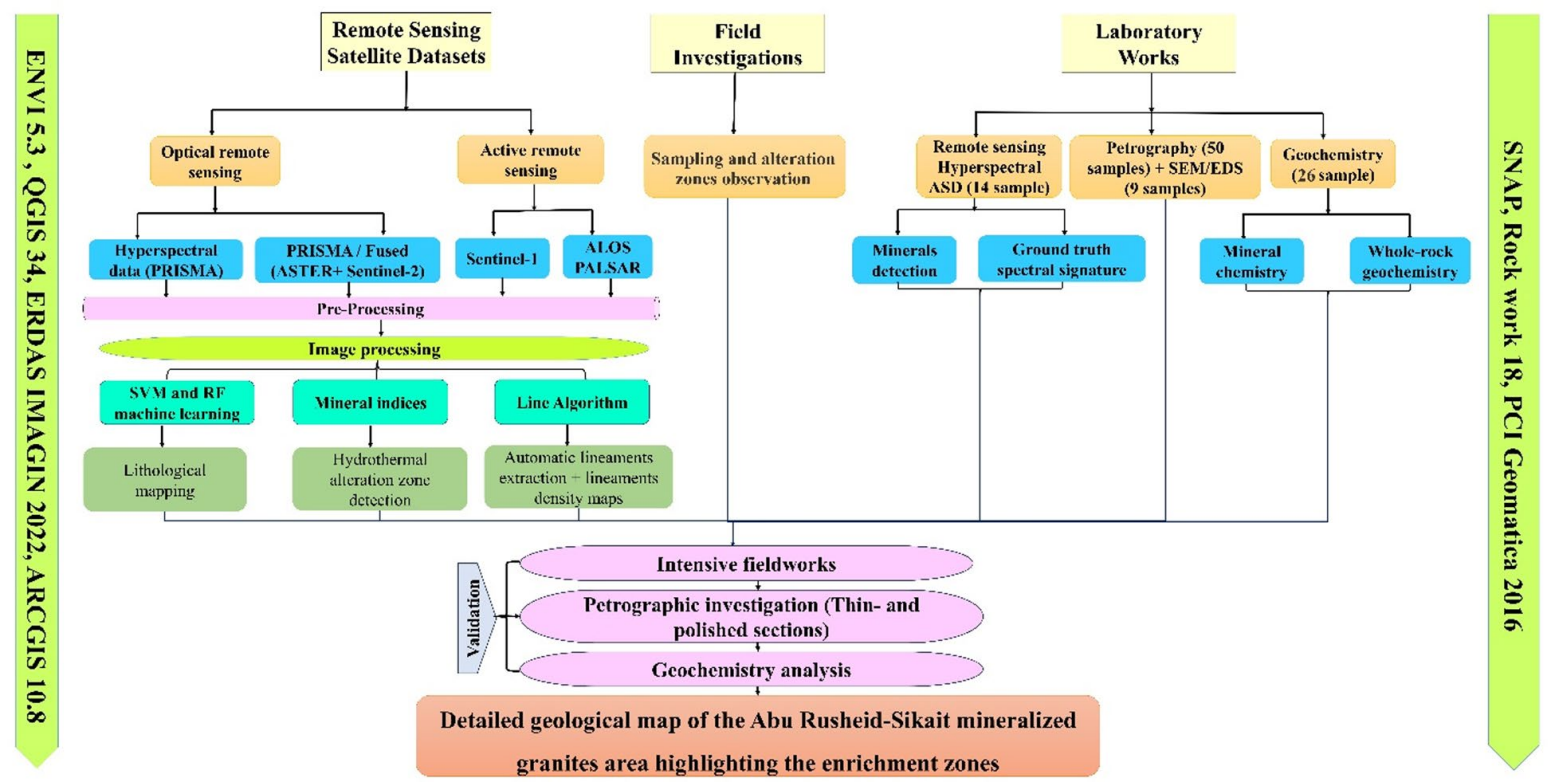
Flowchart of the methodology used in this study.

The detailed materials and methods are provided in Supplementary 2. As illustrated in Fig. 4, this work adopted an integrated methodology that combines remote sensing, field investigations, petrographic description, and geochemical analyses.

## Results

### Remote sensing

#### Automatic lithological mapping based on Hyperspectral PRISMA data

**Fig. 5:**
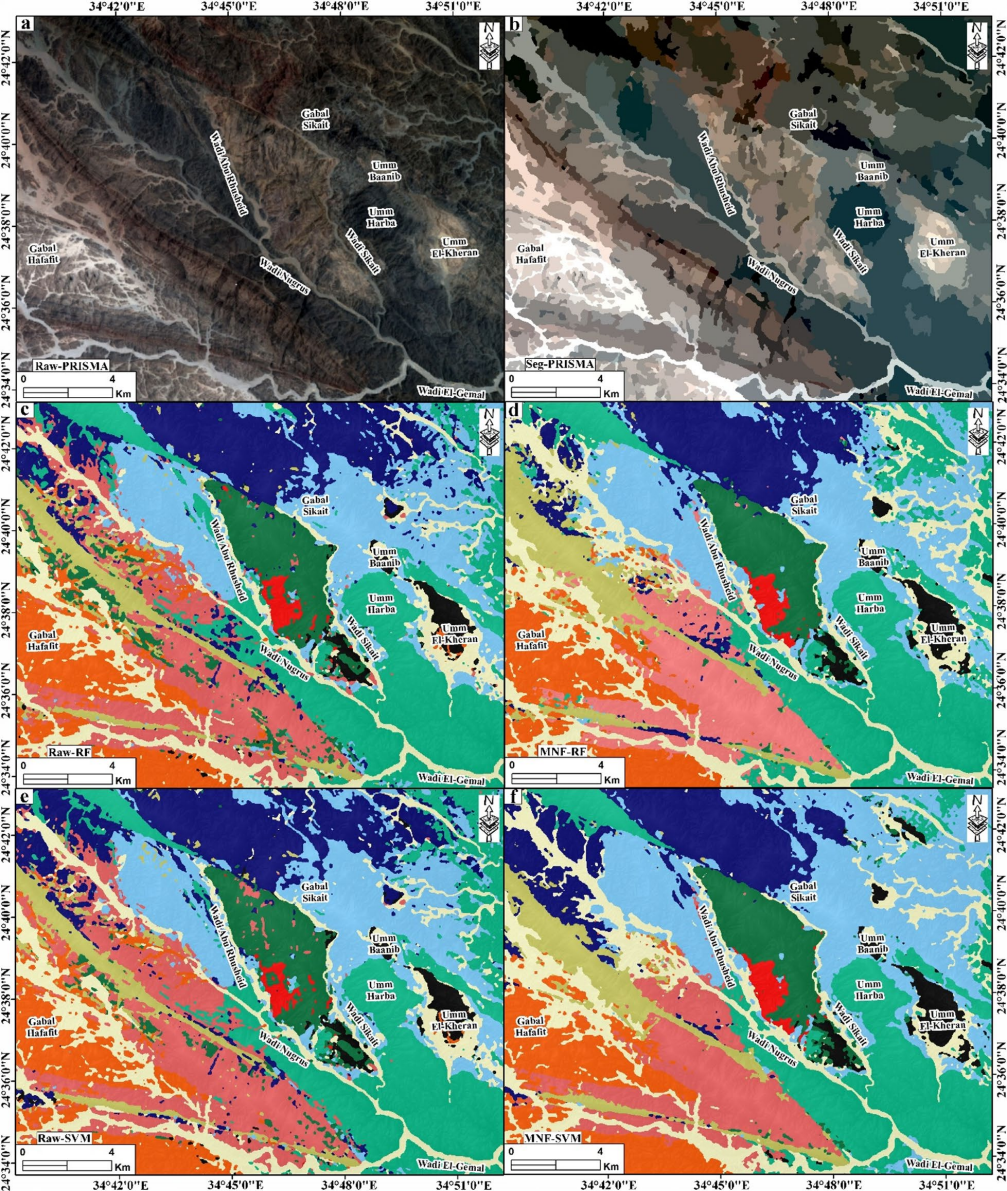
(**a**) Hyperspectral PRISMA raw data displayed as an RGB composite (b30, b20, b9). (**b**) Semantic segmentation of the PRISMA data. Lithological classification map generated using the Random Forest (RF) algorithm applied to the raw PRISMA spectral bands (**c**) and Minimum Noise Fraction (MNF) transformed PRISMA data (**d**). Lithological classification map generated using the Support Vector Machine (SVM) algorithm applied to the raw PRISMA spectral bands (**e**) and MNF-transformed PRISMA data (**f**). Created by QGIS Desktop 3.36.3 software; https://qgis.org/project/visual-changelogs/visualchangelog336/ and ArcGIS Desktop 10.8. https://www.esri.com/en-us/arcgis/products/arcgis-desktop/overview.

The automatic lithological mapping of various rock units has recently utilized pixel-based classification with several machine learning techniques^5,19,20^. This study utilized 269 training datasets, comprising 35,784 pixels (Supplementary 2), to establish two types of artificial intelligence (AI) machine learning techniques: random forest (RF) and support vector machine (SVM). The training datasets were derived from field observations of ground truth ASD spectral signatures, which were classified into ten distinct classes (Supplementary 3 − 1), hyperspectral PRISMA band combination (b30, b20, b10; Fig. 5a), and its corresponding semantic segmentation (Fig. 5b). To enhance the accuracy of lithological identification, we employed the Minimum Noise Fraction (MNF) approach instead of the original hyperspectral PRISMA data when applying both SVM and RF classifiers (Supplementary 3 − 1). The training datasets were split into 20% for testing and 80% for training (Supplementary 2). Moreover, to ensure the model performance, we adjust the best hyperparameters that maximized the Kappa coefficient on a 20% independent validation subset. For the Random Forest (RF) classifier, we optimized the model with 100 estimators using Gini impurity as the splitting criterion and the square root of the total bands for feature selection, as accuracy plateaued beyond this complexity. For the Support Vector Machine (SVM), we utilized a Radial Basis Function (RBF) kernel with a penalty parameter C = 1.00, gamma = 0.05 and probability estimates enabled, which provided the most robust separation of the non-linear spectral boundaries between lithological units without overfitting.

##### Random Forests (RF)

Random forest classification is a machine-learning technique that utilizes decision trees to generate predictions from a given dataset^68^. In this method, the number of predictor variables used is called the number of trees, and this number represents the height of the tree. The number of random sampling points derived from the study plots represents the total number of trees (ntree). The RF method has been recognized for its effectiveness in the field of earth sciences, particularly in the context of automatic geological mapping^4,19,20^. In this study, the RF classifier has been applied to the original bands of the hyperspectral PRISMA data and the transformed MNF dataset, which contains enhanced spectral responses of various lithological units. It is anticipated that this will result in improved classification accuracy compared to that achieved with the original datasets. The RF classifier applied to the PRISMA original data gives an overall accuracy of 84.79%, a Kappa coefficient of 82.64%, and a mean F1-score of 82.57% (Fig. 5c; Supplementary 3 − 1). On the other hand, the enhanced MNF-PRISMA dataset gives an overall accuracy of 88.13%, a Kappa coefficient of 86.37%, and a mean F1-score of 87.86% (Fig. 5d; Supplementary 3 − 1). Ophiolitic mélange, ophiolitic metagabbros, and alkali feldspar granites in both datasets show excellent performance values > 88% (Supplementary 3 − 1). Zinnwaldite-muscovite granites, garnet-muscovite granites, Li-rich pegmatitic granites, and Wadi deposits exhibit the best performance values in the enhanced MNF-PRISMA data, ranging from 91.25% to 92.75% (Supplementary 3 − 1). Tonalites-granodiorites have a good performance in both datasets, with an F1-score up to 86.98%, while gneisses rocks and biotite granites have moderate to good accuracy with an F1-score up to 82.16% and 79.94%, respectively (Supplementary 3 − 1).

##### Support Vector Machine (SVM)

Support Vector Machines (SVM), developed by Cortes and Vapnik^69^ as a supervised machine learning technique, classify data by constructing an optimal decision boundary (hyperplane) that maximizes the margin between distinct classes. This approach identifies support vector data points closest to the hyperplane to define parallel class-separating boundaries, ensuring robust generalization to new data. In geology, the RBF-SVM algorithm (using a radial basis function kernel) has gained prominence for automated lithological mapping due to its efficiency in handling high-dimensional spectral datasets^5,19,20,70,71^. The precision of classification and the efficacy of SVM training depend on selecting the right kernel function. The radial basis function (RBF) kernel has been demonstrated to possess commendable interpolation capabilities^72^, and thus it was utilized in the present study. In this work, the hyperspectral PRISMA-SVM classified image was used to identify the widely exposed lithological units in the study area (Fig. 5e, f).

The overall accuracy and Kappa coefficient for PRISMA-SVM classified images are 84.28% and 81.96%, respectively, with a mean F1-score of 82.16% (Supplementary 3 − 1). The transformed MNF of PRISMA gives an overall accuracy of 89.02%, a Kappa accuracy of 87.41%, and a mean F1-score of 88.61% (Supplementary 3 − 1). In SVM-classified images, the highest accuracy results are represented by the ophiolitic mélange, ophiolitic metagabbros, tonalites-granodiorites, and alkali feldspar granites with F1-scores of 90.37%, 89.18%, 88.97%, and 88.26%, respectively (Fig. 5e; Supplementary 3 − 1). Zinnwaldite-muscovite granites exhibit the lowest accuracy, with an F1-score of 66.86% (Fig. 5e; Supplementary 3 − 1). The SVM applied to the enhanced MNF of PRISMA data yields excellent results for all classes, with an F1-score ranging from 86.63% to 93.19%, except for the gneiss rocks, for which it achieves a high-performance F1-score of 81.83% (Fig. 5f; Supplementary 3 − 1).

#### Mineral mapping

**Fig. 6:**
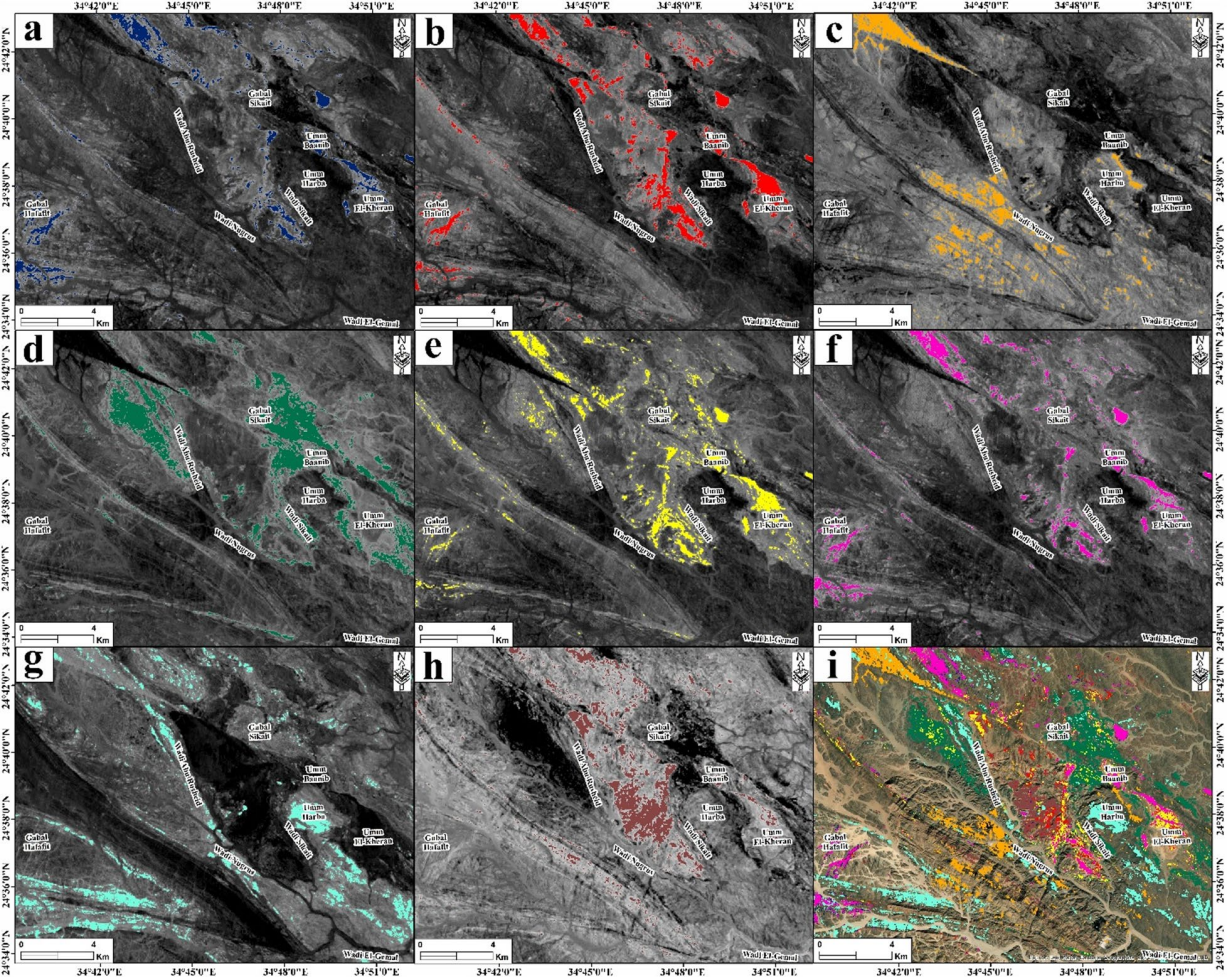
Greyscale hyperspectral PRISMA alteration and mineral indices in the Abu Rusheid-Sikait area. (a) PRISMA index (b189 + b201), highlighting phyllic alteration zones in blue. (b) Muscovite index (b201/b194) highlighting the muscovite-rich zones in granitic rocks with red color. (c) PRISMA index (b189/b132) emphasizing the ferrous silicate (biotite, chlorite, amphibole) alteration zones in orange. (d) Argillic alteration index (b132 + b194/b189), emphasizing alteration zones enriched in alunite, kaolinite, and pyrophyllite in green. (e) Kaolinite alteration index (b132/b189)×(b210/b194), detecting kaolinite-rich alteration zones in yellow. (f) PRISMA index (b201/b194) × (b132/b194) emphasizes the hydroxyl-bearing alteration zones with magenta. (g) Propylitic alteration index (b201 + b220)/b211detecting alteration zones rich in chlorite, epidote, and carbonate minerals in cyan. (h) Ferrugination index (b132/b33 + b33/b21) emphasizes the distinct alteration zones rich in hematite and goethite with brown. (i) Integrated PRISMA mineral indices map, illustrating the spatial distribution of eight mineral types derived from hyperspectral PRISMA data across the Abu Rusheid–Sikait area. area. Created by ENVI v. 5.3 software; https://www.l3harrisgeospatial.com/Software-Technology/ENVI, which is mainly utilized for image processing and ArcGIS Desktop 10.8. https://www.esri.com/en-us/arcgis/products/arcgis-desktop/overview.

The spectral band index is one of the most powerful imaging techniques for hydrothermal alteration mapping^4,^^5,^^20,^^73^. The presence of high digital number values within a given scene is indicative of spectral signatures that are analogous to those of the specific materials for which the values were designed to map^4,^^5,^^20,^^73,^^74^. In this study, different mineral indices were applied to the hyperspectral PRISMA dataset to detect the hydrothermal alteration zones affecting the various rock types of the Abu Rusheid-Sikait area (Fig. 6). The hyperspectral PRISMA mineral indices successfully detect different hydrothermal alteration zones in the mineralized granites (Fig. 6).

**Fig. 7:**
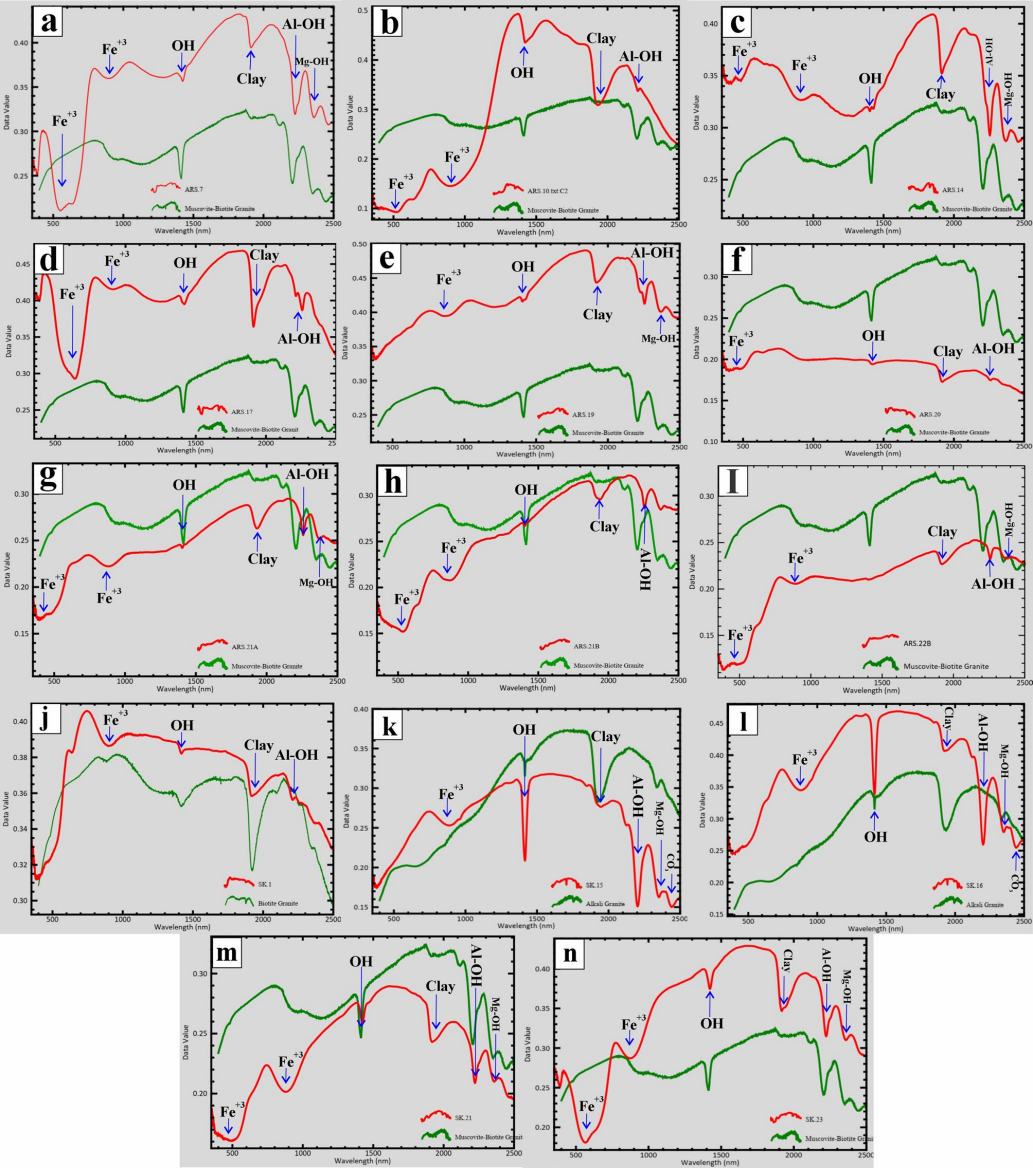
ASD measurements of zinnwaldite-muscovite granites and biotite granites in the Abu Rusheid-Sikait granites, showing that the spectral signature compared to the ASTER library ENVI. The key features labeled as Fe³⁺, OH, clay, Al-OH, and Mg-OH indicate mineral identification based on their absorption characteristics. Created by ENVI v. 5.3 software; https://www.l3harrisgeospatial.com/Software-Technology/ENVI.

A greyscale PRISMA index (b189 + b201)/b194 well emphasizes the phyllic alteration zones in the area with blue color (Fig. 6a). The spectra of phyllic alteration zones are characterized by the presence of sericite, muscovite, and/or illite reflectance spectra, which exhibit an intense Al-OH absorption feature (Supplementary 3 − 2). This feature is typically centered between 2185 and 2225 nm (Fig. 7; Supplementary 3 − 2), which coincides with PRISMA band 194^4,^^5,^^62,^^20,^^29^. The PRISMA (b201/b194) index visualizes the abundance of muscovite with a red color (Fig. 6b) due to its strong absorption in band 194, while it has a high reflectance in PRISMA band 201^75^. Muscovite is an important mineral in this study since it serves as an indicator of hydrothermal alteration processes^76^ due to its frequent secondary formation in the granites. The hematization of ferrous silicates like biotite and chlorite has been identified with an orange color (Fig. 6d) using the PRISMA index (b189/b132).

The argillic alteration zones show typical spectral features of minerals like kaolinite, alunite, and pyrophyllite, which exhibit distinctive absorption features at 2165 nm (Fig. 7; Supplementary 3 − 2), corresponding to the PRISMA band 189^74^. Argillic and kaolinite alterations were emphasized with green and yellow colors, respectively (Fig. 6d, e), based on the PRISM indices (b132 + b194/b189) and (b132/b189) × (b210/b194), respectively^77,78^. Alteration minerals with hydroxyl groups (OH), such as montmorillonite, are distinguished by a distinctive spectral absorption feature between 2185 and 2225 nm (Fig. 7; Supplementary 3 − 2), observed in the PRISMA band 194 region. This phenomenon results in a notable increase in reflectance in the PRISMA bands 132 and 201, while the reflectance in the band 194 region remains relatively low. Therefore, the PRISMA index (b201/b194)×(b132/b194)^5,70^ has been used to detect hydroxyl-bearing minerals in the area, which is mapped with magenta color (Fig. 6f).

The propylitic alteration reflectance spectra are distinguished by the presence of Fe, Mg-OH absorption features, and CO_3_ features, which are attributed to chlorite, epidote, and carbonate minerals^74^. Absorption features are present in the 2330 nm region (Fig. 7; Supplementary 3 − 2), which coincides with the PRISMA band 210^5,20,68,78^. The PRISMA index (b201 + b219)/(b210) has thus been used to visualize this alteration zone, which is marked by a cyan color (Fig. 6g). Fe-rich alteration minerals formed during ferrugination and represented by hematite and goethite are distinguished by a distinctive spectral absorption feature at ca. 600 and 900 nm (Fig. 7; Supplementary 3 − 2) observed in the PRISMA bands 21 and 33 regions. This phenomenon results in a notable increase in reflectance in the PRISMA bands 132 and 33 regions. Therefore, they were detected using the PRISMA index (b132/b33 + b33/b21) with a brown color (Fig. 6h). The eight identified alteration zones in the area are shown in Fig. 6i.

#### Automatic lineaments extraction

**Fig. 8:**
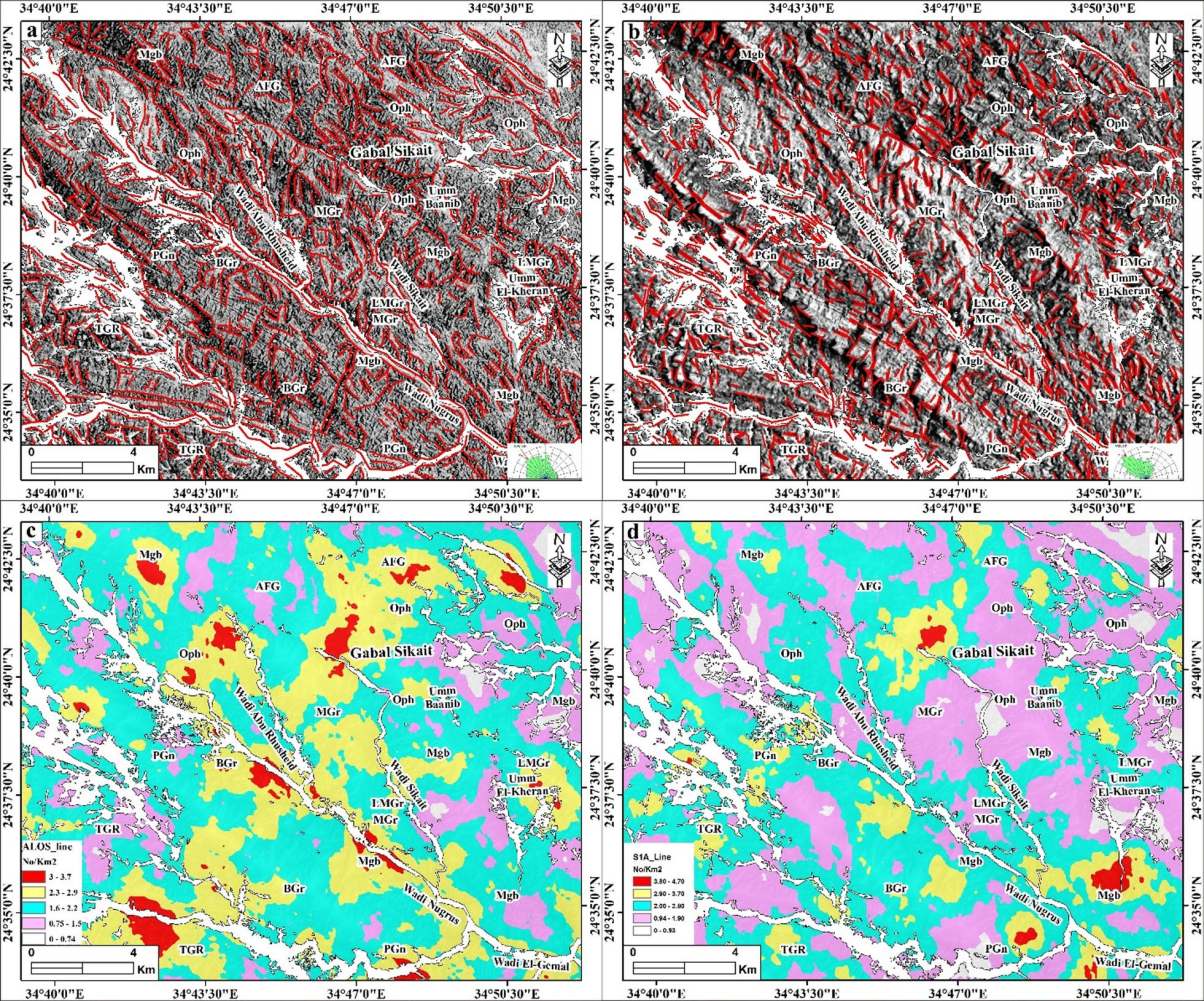
(**a**, **b**) Lineaments extraction and rose diagrams based on ALOS PALSAR and Sentinel-1 A, respectively. (**c**,** d**) Lineaments density map of ALOS PALSAR and Sentinel-1 A, respectively. We observed that ALOS PALSAR shows the highest density concentration between different rock types in the study area. Abbreviations: Li-pegmatitic granites (LPGr), garnet-muscovite granites (GMGr), alkali feldspar granites (AFG), biotite granites (BGr), tonalites-granodiorites (TGR), metagabbros (Mgb), ophiolitic mélange (Oph), gneisses rock (Gn), Wadi deposits (WD), and zinnwaldite-muscovite granites (LMGr). Created by ArcGIS Desktop 10.8. software; https://www.esri.com/en-us/arcgis/products/arcgis-desktop/overview, ENVI v. 5.3. software; https://www.l3harrisgeospatial.com/Software-Technology/ENVI, Geomatica PCI software and RCOKWORK v. 18 software; https://www.rockware.com/product/rockworks/.

Automated lineament detection offers a faster and more objective approach to geologic mapping compared to traditional manual (visual) interpretation, which is more dependent on the experience of the analyst. The rapid extraction of lineaments is essential for geologic mapping and identifying the alteration zones, which are often associated with hydrothermal ore deposits close to or within the fault/fracture zones^4,5,9,10,20^. Figure 8a, b shows the results of lineaments extracted from the ALOS PALSAR and Sentinel-1 datasets in the Abu Rusheid-Sikait area. The predominant surface structural lineament directions in the study area are NW-SE, N-S, and E-W (Fig. 8a, b). Subsequently, these data were exported to ArcGIS version 10.8 to perform further analysis, including lineament density mapping using a line density module (Fig. 8c, d).

This module depends on the frequency of the lineaments per unit area (number/km^2^). Remarkably, the lineaments and their densities are highly concentrated in some specific zones in the study area, like in the western part of Gabal Sikait in between the garnet-muscovite granites, the ophiolitic mélange, and the alkali feldspar granites (Fig. 8c, d). Moreover, they are concentrated along the Wadi Abu Rusheid and Wadi Nugrus, marking the contacts between gneisses rocks and ophiolitic units and the granitic rocks (Fig. 8c, d). Additionally, they are highly concentrated in the Umm El-Kheran area and in the southern part of the mapped area, within the tonalites-granodiorites (Fig. 8c, d).

### Petrography

#### Tonalites-granodiorites

**Fig. 9:**
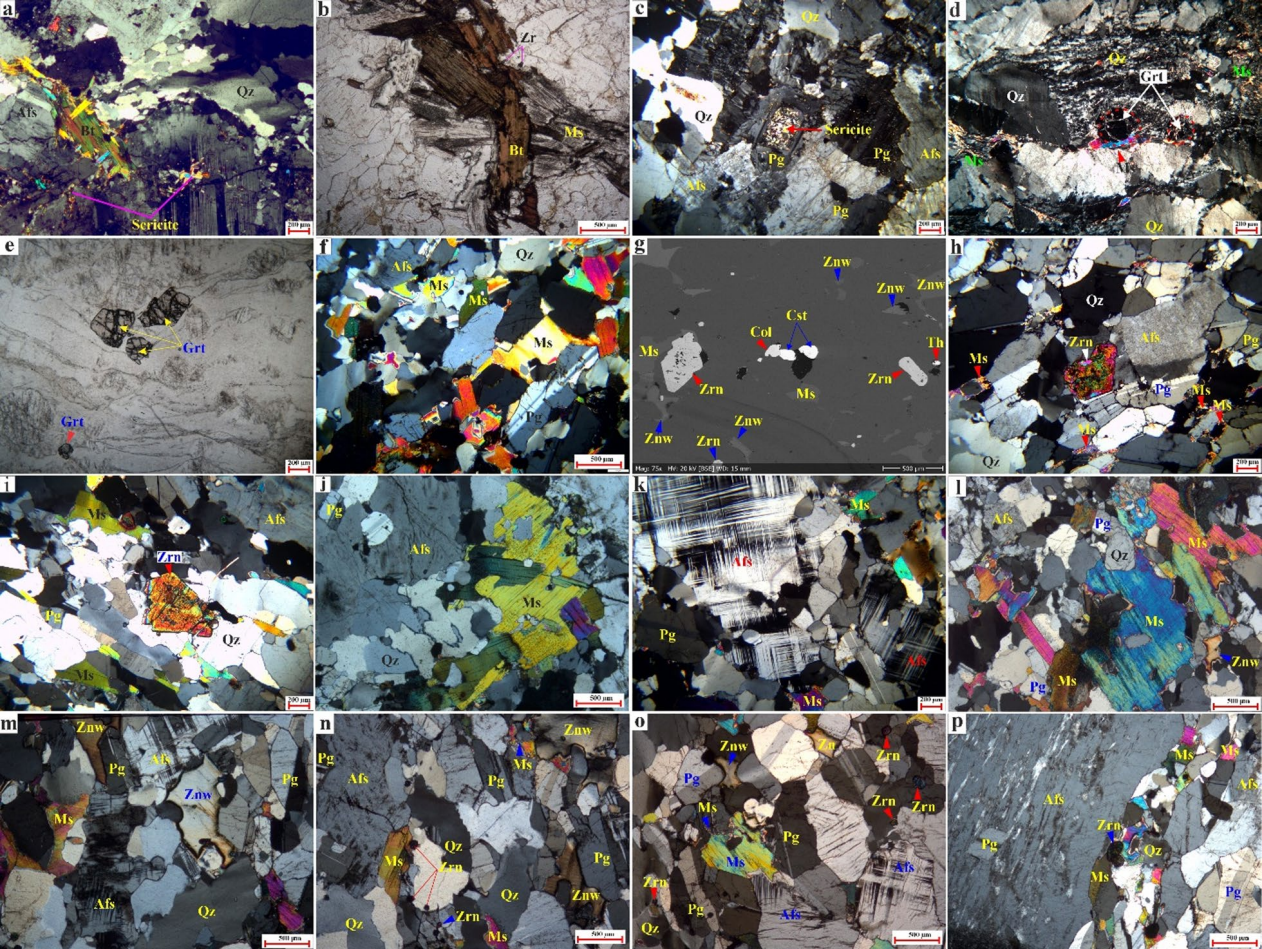
Photomicrographs from different granitic rocks of the Abu Rusheid-Sikait area showing mineralogical and textural variations. (**a**) Deformed magmatic assemblage (Pg, Qz, Bt, Afs) showing undulose extinction and marginal recrystallization of quartz into micro-shear zones. (b-c) Biotite granites: (**b**) Intergrowth of primary biotite and muscovite flakes. (**c**) Magmatic assemblage featuring concentrically zoned plagioclase with sericitized cores. (d–f) Garnet-Muscovite Granites: (**d**) Deformed quartz-muscovite ribbons wrapping preserved garnet grains. (**e**) Fragmented subhedral garnet. (**f**) Weakly foliated equigranular assemblage of white mica, quartz, and feldspars. (g–i) Accessory Minerals: (**g**) Early inclusions of zircon, columbite, and cassiterite in quartz. (**h**) Large zircon crystal associated with fine secondary muscovite. (**i**) Broken, likely inherited zircon within a foliated matrix. (j–p) Zinnwaldite-Muscovite and Li-Pegmatite Granites: (**j**) K-feldspar hosting plagioclase inclusions alongside late anhedral quartz and muscovite. (**k**) Microcline associated with quartz, plagioclase, and interstitial muscovite. (l, n) Weakly foliated fabrics defined by aligned plagioclase, quartz, and alkali feldspar with intergrown muscovite and zinnwaldite. (m, o) Isotropic textural equivalents of the foliated units. (p) Porphyritic pegmatite texture displaying subhedral alkali feldspar in a fine-grained matrix. Abbreviations: Albite (Ab), quartz (Qz), alkali feldspar (Afs), plagioclase (Pg), muscovite (Ms), zinnwaldite (Znw), biotite (Bt), chlorite (Chl), zircon (Zrn), garnet (Grt), columbite (Col), cassiterite (Cst), thorite (Th), xenotime (Xtm), kasolite (Kls), fluorite (Fl), and monazite (Mnz). Figures 9 and 10 were taken by a polarizing microscope at the Geology Department, Kafrelsheikh University, and the Institute of Applied Geosciences at Karlsruhe Institute of Technology in Germany.

Tonalites-granodiorites exhibit a medium- to coarse-grained texture and are characterized by a color range from white to whitish grey, displaying an equigranular texture (Fig. 9a). The main minerals include plagioclase (45–50 vol%), quartz (25–30 vol%), K-feldspar (10–15 vol%), biotite (ca. 5 vol%), and minor amphibole (ca. 2 vol%) (Fig. 9a). Alteration minerals identified include chlorite, sericite, muscovite, and kaolinite (Fig. 9a). Furthermore, accessory minerals consist of Fe-Ti oxides, zircon, garnet, sphene, and apatite. Plagioclase is the predominant mineral, presenting as medium- to coarse-grained, subhedral to anhedral tabular crystals (Fig. 9a). A significant degree of alteration is observed in the core, where plagioclase transforms into sericite and kaolinite (Fig. 9a). Some large plagioclase crystals exhibit normal zoning. Quartz is found as medium- to coarse-grained, anhedral crystals characterized by wavy extinction, resulting from deformation along micro-shear zones (Fig. 9a). K-feldspar manifests as an anhedral to subhedral crystals situated within the interstices between quartz and plagioclase, comprising microperthitic orthoclase (Fig. 9a). Biotite is observed as medium- to coarse-grained, brownish flakes and aggregates, with a tendency for partial or complete alteration to chlorite (Fig. 9a). Structurally, the flakes display distinct bending and kinking of cleavage lamellae, consistent with the deformation observed in quartz, and show a tendency for partial or complete alteration to chlorite along these structural weaknesses. Amphiboles can be found as tiny green anhedral lath-shaped crystals, which have undergone partial alteration to Fe oxides and chlorite. Muscovite is present in the form of minute flakes or aggregates within the interstitial spaces of other rock-forming minerals. Zircon appears as fine-grained subhedral crystals or as tiny inclusions within biotite and amphibole.

#### Biotite granites

Biotite granites exhibit a texture that ranges from medium to coarse-grained granularity (Fig. 9b, c). The essential mineral constituents consist of K-feldspar (45–50 vol%), quartz (20–30 vol%), plagioclase (20–25 vol%), and biotite (ca. 4 vol%) (Fig. 9b, c). The alteration minerals are represented by chlorite, sericite, and kaolinite (Fig. 9b, c). Zircon and Fe-Ti oxides represent the main accessory minerals (Fig. 9b). K-feldspar is manifested as perthitic subhedral to anhedral crystals interspersed with albite stringers (Fig. 9c). Some K-feldspar crystals show inclusions of quartz and plagioclase. Plagioclase is predominantly identified as subhedral-tabular crystals, with a subset of grains exhibiting replacement by sericite within their cores (Fig. 9c). Quartz occurs as medium to coarse-grained, anhedral crystals, occasionally exhibiting triple-junction grain boundary contacts (Fig. 9c). Biotite is identified as fine to coarse-grained, subhedral flakes characterized by a brownish hue (Fig. 9b). A fraction of biotite crystals has experienced weak to extensive alteration into chlorite and iron oxides along their cleavage planes. Zircon is observed as subhedral to euhedral inclusions within biotite.

**Fig. 10:**
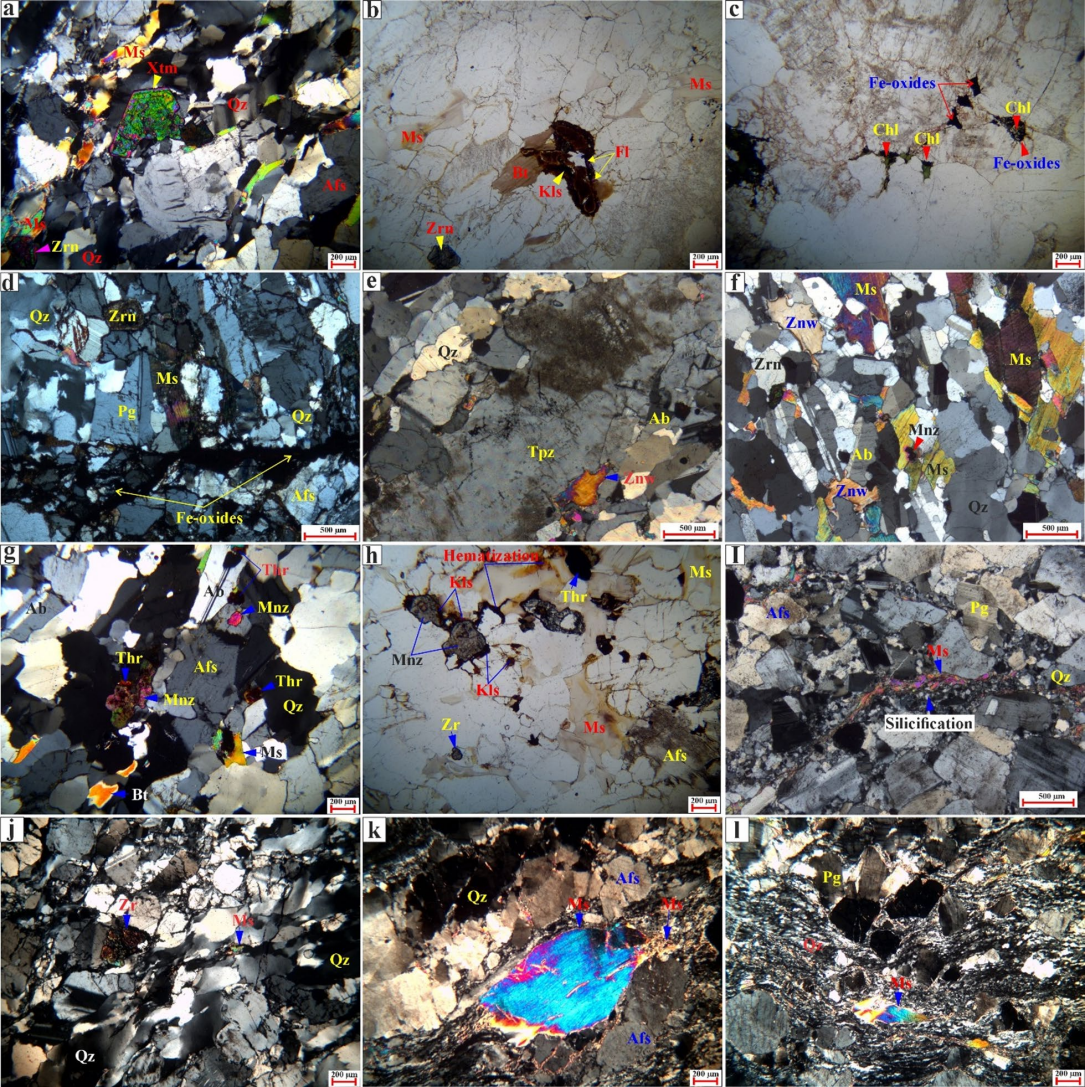
Photomicrographs from different granitic rocks of the Abu Rusheid-Sikait area showing mineralogical and textural variations. (a) Heterogranular assemblage of plagioclase, quartz, muscovite, and perthitic K feldspar. Xenotime occurs as a large, fragmented crystal (likely inherited). Zircon is a frequent inclusion in the magmatic phases; (b) Relic of a primary radioactive mineral (likely metamict zircon) replaced by secondary brown Kasolite and transparent fluorite in zinnwaldite-muscovite granites. (c) Fe oxide occurs as an anhedral interstitial phase, partially intergrown with chlorite (likely replacing primary biotite) in biotite granites. (d) Texturally late hematite veinlets transect the magmatic assemblage of quartz, plagioclase, muscovite, and zircon, reflecting the deformation and ferrugination alteration in zinnwaldite-muscovite granites. (e) Subhedral large topaz crystal associated with zinnwaldite and albite; (f) Large flakes of muscovite hosted by monazite and associated with medium subhedral zinnwaldite crystals; (g) large anhedral monazite crystals rimmed by thorite in the interstitial spaces between alkali feldspar and quartz, small crystals as inclusions in quartz, small to medium flakes of mica (muscovite and biotite), and small disseminated thorite; (h) secondary uranium mineral (kasolite) rimmed large anhedral monazite associated with hematization and small disseminated thorite crystals; (i) secondary quartz enrichment (silicification) associated with secondary muscovite flakes; (j) Quartz exhibits grain elongation, undulose extinction, subgrain formation, and dynamic recrystallization, with shear bands and microfractures defining the deformation fabric (k); a large, elongated porphyroclasts (mica) including microfractures and marginal alteration, wrapped by a fine-grained quartz matrix, forming a well-developed porphyroclastic–mylonitic fabric, reflecting intense shear deformation under brittle-ductile conditions. (l) a fine-grained, sheared quartz-feldspar matrix enclosing fractured and rotated porphyroclasts. High-strain zones are characterized by aligned ribbons of minerals with high-order interference colors, recording intense ductile shearing with localized brittle overprint in zinnwaldite-muscovite granites. For abbreviations (see Fig. 9).

#### Garnet-muscovite granites

The garnet-muscovite granites are characterized by a medium- to coarse-grained texture and exhibit weak shearing. Their essential composition is predominantly K-feldspar (40–45 vol%), quartz (25–30 vol%), and plagioclase (12–15 vol%), and contains abundant muscovite (4–10 vol%), garnet (5–15 vol%), and trace amounts of biotite (ca. 1 vol%) (Fig. 9d, e). Other accessory minerals identified within these granites include zircon, xenotime, and Fe-Ti oxides. K-feldspar is observed in the form of medium to coarse-grained, anhedral to subhedral crystals of orthoclase microperthite or microcline (Fig. 9d, e), which are strongly sericitized in part of the samples (Fig. 9d). Quartz is present as a medium- to coarse-anhedral grain with wavy extinction or as fine-grained, strongly recrystallized grain aggregates defining the weak foliation. Texturally late, fine-grained quartz fills small, irregular veinlets (Fig. 9d). Plagioclase is present as subhedral to anhedral tabular and elongated crystals. Muscovite has two generations: primary crystals of large, subhedral flakes and secondary, fine aggregates that form along the margins of late quartz (Fig. 9d). Garnet grains occur as medium-grained, subhedral crystals and exhibit inclusions of quartz and zircon, reflecting their magmatic origin. In addition, garnet forms grain aggregates and veins (Fig. 9d, e). Zircon is identified as fine- to medium-grained subhedral to euhedral crystals, particularly as inclusions in garnet and muscovite.

#### Zinnwaldite-muscovite granites

**Fig. 11:**
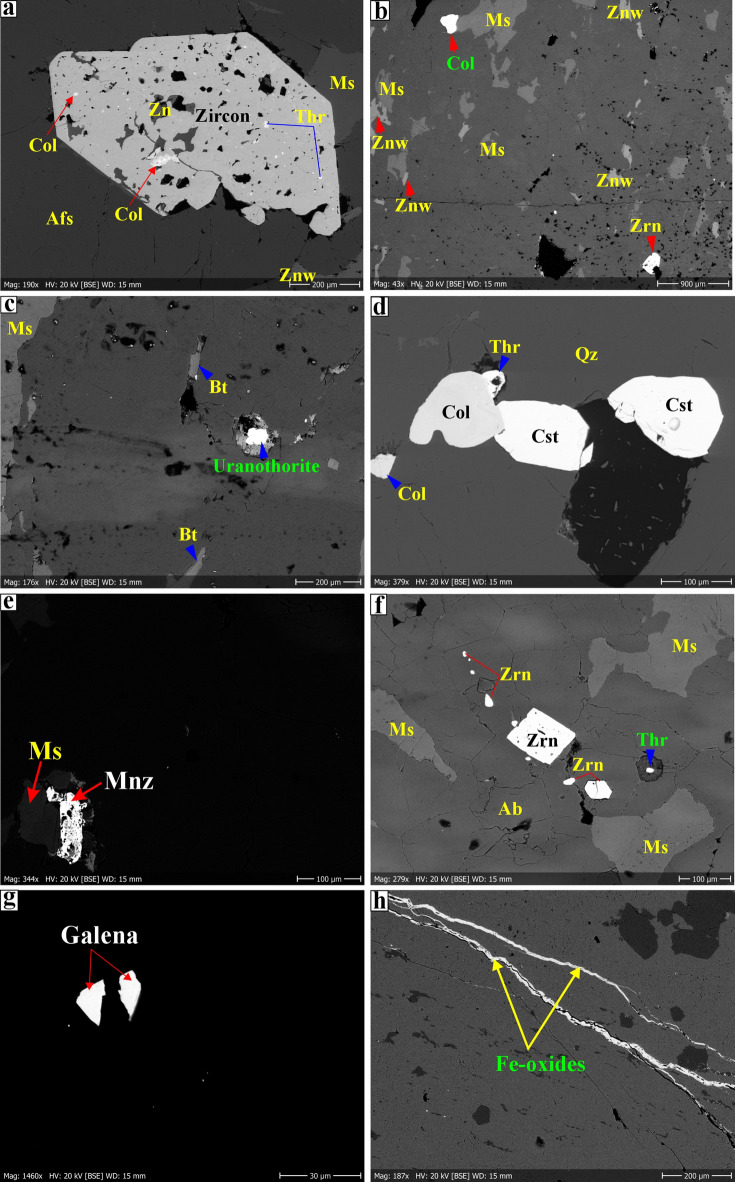
Scanning Electron Microscopy images (SEM) of rare-metal minerals in the Abu Rusheid-Sikait area. **a**) Inclusion of euhedral zircon (Zrn) in alkali feldspar (Afs). The poikilitic zircon contains abundant inclusions of columbite (Col), thorite (Thr), and quartz (Qz); b) Individual grains of zircon (Zrn) and columbite (Col), associated with muscovite (Ms) and zinnwaldite grains (Znw); **c**) Thorite inclusion in biotite; **d**) Cassiterite (Cst) and columbite (Col) inclusion in quartz; **e**) Monazite (Mnz) rimmed by white mica (Ms) next to quartz; **f**) Zircon (Zrn) and thorite (Thr) inclusions in albite and quartz, respectively; **g**) Galena next to quartz; **h**) Hematite veinlets. SEM images were taken at the Department of Mineralogy and Petrology at Karlsruhe Institute of Technology, Germany.

Zinnwaldite-muscovite granites exhibit a medium to coarse grain size and are characterized by a deformed (sheared) texture (Figs. 9f-m and 10a-d). They are composed of K-feldspar (40–45 vol%), quartz (30–35 vol%), plagioclase (10–18 vol%), muscovite, and zinnwaldite (8–15 vol%) as essential minerals (Figs. 9f-m and 10a-d). Zircon is a common accessory phase (ca.1–3 vol%), besides garnet, xenotime, Fe-Ti oxides, columbite, thorite, secondary uranium mineral (kasolite), topaz, fluorite, cassiterite, galena, and monazite (Figs. 9g-i, 10 and 11). K-feldspar is the most abundant mineral, manifesting as subhedral tabular microcline and orthoclase-perthite varieties (string, flame, and braided) (Fig. 9f, h-l). Quartz is present as primary crystals that are frequently strained, exhibiting a characteristic undulose extinction (Fig. 9f, h-l). In some samples, minor secondary quartz fills veins (Fig. 10i). Plagioclase is identified in two distinct textures; the first is characterized by medium to coarse-grained, subhedral tabular crystals exhibiting albite twinning within the matrix (Fig. 9f, h, k), while the second is represented by fine-grained inclusions of euhedral plagioclase embedded within K-feldspar (Fig. 9j). Primary muscovite is present as subhedral to euhedral, medium to coarse-grained crystals that are locally intergrown with biotite (Figs. 9f-l and 10f).

Secondary muscovite forms fine-grained crystal aggregates or veinlets along the grain margins of the feldspars (Fig. 9h). Quartz, K-feldspar, and muscovite define a weak foliation due to plastic deformation (Fig. 9f, i-l). Zinnwaldite is the predominant lithium-bearing mineral in this rock type (Fig. 5l, m). It is characterized by an anhedral, micaceous habit (Fig. 9l, m). In most cases, the fine- to medium-grained zinnwaldite is associated with muscovite (Figs. 9h, l). Zircon with pleochroic haloes is present as inclusions in muscovite or as medium-grained, subhedral to euhedral, prismatic, and zoned crystals (Figs. 9f-l and 10a). It contains inclusions of quartz, radioactive minerals (thorite), and columbite (Fig. 11a). Columbite is either found as an inclusion within zircon or is found in association with other minerals like cassiterite (Fig. 9g). Xenotime forms subhedral crystals with high interference colors (Fig. 10a). Secondary uranium minerals are represented by kasolite, which is associated with fluorite and replaces zircon (Fig. 10b). Furthermore, it may occur as minute dispersions or as micro-fractures that infiltrate and coat the surfaces of hematized joints (Fig. 10b, h). Kasolite is characterized by a brownish-yellow hue with a greasy luster and can be observed in various forms, including radial fibers, aggregates, vein-like structures occupying fractures, or as rounded spheres (Fig. 10b, h). Fe oxide minerals are present as euhedral to subhedral crystals situated in the interstices between other rock-forming minerals or in association with chlorite, likely serving as a replacement for primary biotite. Topaz is identified as euhedral to subhedral crystals in association with zinnwaldite, quartz, and albite (Fig. 10e). Galena is the primary lead mineral that occurs as fine grains (Fig. 11g). Monazite occurs as an inclusion in and at the rims of muscovite with pleochroic haloes (Figs. 10f and 11e). Na metasomatism led to coarsening of perthitic exsolution lamellae of alkali feldspar to patch perthite and migration of albite lamellae into K-feldspar grain boundaries (Fig. 9h-m). A partial replacement of K-feldspar megacrysts by albite is observed locally, with albite displaying inclusions of preserved K-feldspar with corrugated borders (Fig. 9h-m). Typical hydrothermal alteration features of the zinnwaldite-muscovite granite include cloudy and turbid K-feldspar due to sericitization (Fig. 9f-k) and replacement of biotite by chlorite and Fe oxide, and formation of quartz veins (Fig. 10c). Ferrugination alteration is observed by the opaque iron oxides (hematite/goethite) associated with secondary uranium mineral (kasolite), indicating late-stage ferrugination along microcracks (Fig. 11d, h).

#### Li-rich pegmatitic granites

Li-rich pegmatitic granites have coarse to pegmatitic grain sizes of up to 3 cm and are characterized by a granular texture. The mineral composition predominantly consists of K-feldspar (50–55 vol%), quartz (20–25 vol%), and plagioclase (10–15 vol%), as well as muscovite and zinnwaldite (ca. 4–8 vol%) (Fig. 9n, o). Accessory minerals commonly identified within these granites include garnet, zircon, Fe-Ti oxides, allanite, and sphene (Fig. 9o). K-feldspars are primarily represented by orthoclase perthite and microcline perthite (Fig. 9n, o). They appear to be embayed and sometimes enclose albite crystals. K-feldspar crystals demonstrate minimal alteration to sericite and show signs of deformation. Typically, microcline is present as coarse anhedral crystals that contain albite lamellae, thereby forming string perthitic textures (Fig. 9n, o). Plagioclase displays medium to coarse-grained crystals with albite twining (Fig. 9n, o). Quartz exhibits medium-to-coarse-grained and anhedral, occupying the interstitial spaces between the plagioclase and K-feldspar (Fig. 9n, o). Muscovite displays medium- to coarse-grained primary flakes alongside fine-grained secondary crystals (Fig. 9n, o). Zinnwaldite is characterized by a micaceous habit, high relief, and forms medium- to coarse-grained anhedral crystals in the interstices of larger feldspar crystals (Fig. 9n, o). Zircon is found as the main accessory mineral, appearing as individual euhedral to subhedral crystals and as inclusions within quartz, muscovite, and garnet (Fig. 9n, o).

#### Pegmatite veins

Pegmatite veins are very coarse-grained rocks with pink to pinkish-white colors (Figs. 2a and f and 3g). They are composed mainly of K-feldspar, quartz, plagioclase, and muscovite (Fig. 9p). Zircon, monazite, thorite, and columbite are common accessory minerals. K-feldspar is the main mineral and occurs as subhedral to anhedral coarse-grained crystals with different types of perthitic exsolution, including patch and flame types (Fig. 9p). Some K-feldspar crystals contain plagioclase inclusions (Fig. 9p). Plagioclase occurs as subhedral medium crystals with albite twining (Fig. 9p). Quartz displays fine- to medium-grained crystals with undulose extinction (Fig. 9p). Muscovite occurs as anhedral crystals among other rock-forming minerals (Fig. 9p). The most common accessory mineral is zircon (Fig. 9p) which is found as inclusions in feldspars, muscovite, and quartz, or as isolated interstitial subhedral crystals (Fig. 9p).

### Mineral chemistry

Representative EPMA analyses of feldspar, biotite, and zircon have been conducted on the studied biotite granites and zinnwaldite-muscovite granites. Meanwhile, muscovite, zinnwaldite, columbite, and topaz were analyzed only in the zinnwaldite-muscovite granites, due to their abundance in this rock type.

#### Feldspars

**Fig. 12:**
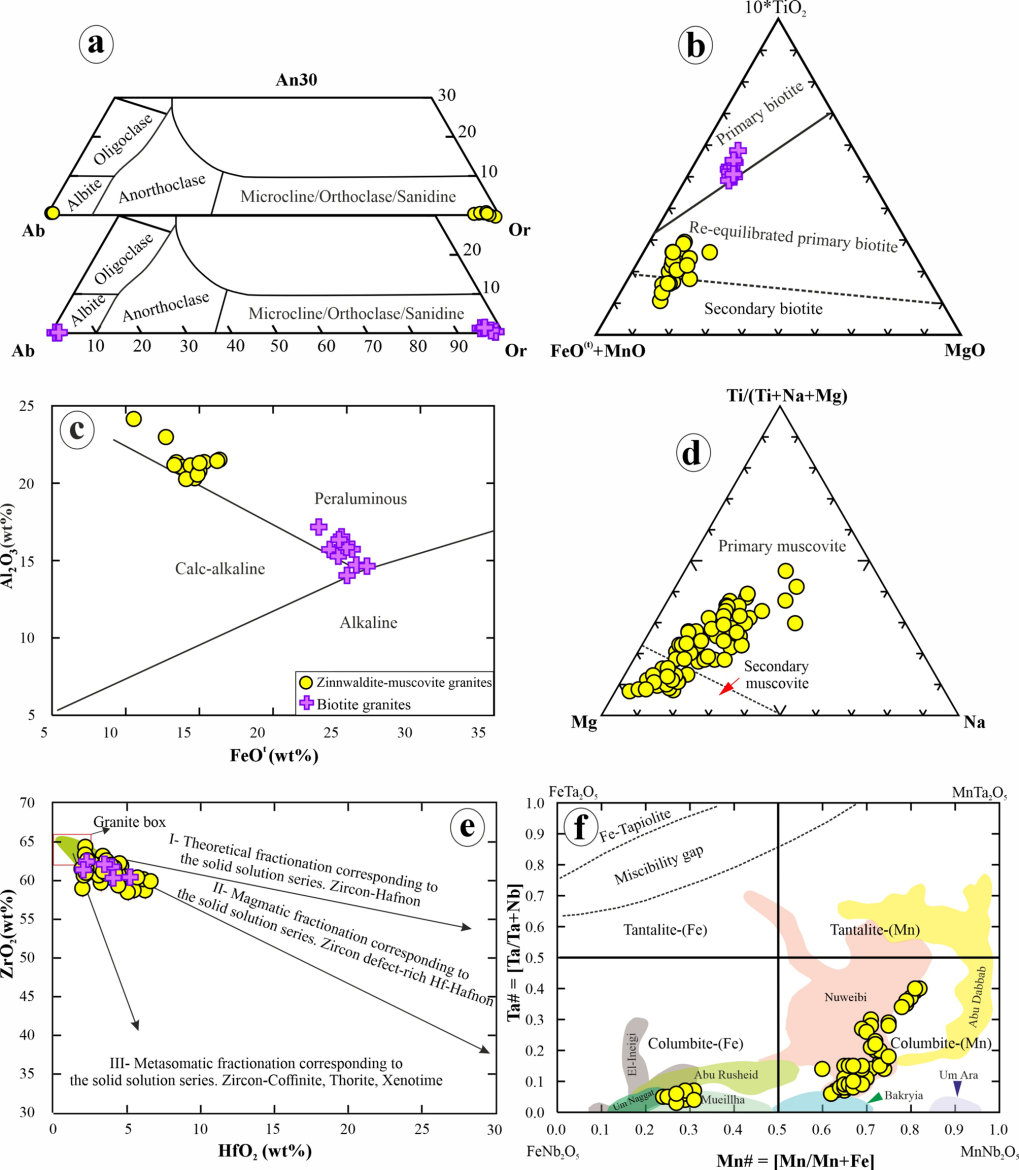
(**a**) Feldspar composition in the An-Ab-Or diagram. (**b**) TiO_2_-(FeOt + MnO)-MgO ternary diagram for classification of biotite (after Nachit et al.^74^. (**c**) FeO^t^ versus Al_2_O_3_ discrimination diagram for biotite^75^. (**d**) Compositional fields for primary and secondary muscovite in the ternary diagram of Miller et al.^76^. (**e**) Binary relation between ZrO_2_ and HfO_2_ of zircon from the studied Abu Rusheid-Sikait granites, the granite box, shows trends and data of zircons from the Egyptian rare metal granites^78^. (**f**) Plot of Ta# [Ta/(Ta + Nb)] versus Mn# [Mn/(Mn + Fe)] ratios of columbite group minerals from the Abu Rusheid-Sikait granites in comparison with others from rare metal granitoids in the Eastern Desert of Egypt^79,80^. Figs. from 12 to 15 have been drawn by the CorelDRAW program version 2022.

EPMA analyses of feldspar minerals (K-feldspar, 51 points, and plagioclase, 76 points) from the studied biotite granites and zinnwaldite-muscovite granites, along with their calculated chemical formulae, are listed in Supplementary 5. The analyzed K-feldspar in zinnwaldite-muscovite granites and biotite granites is similar in composition, with an orthoclase content of 94 to 98 mol% (Fig. 12a; Supplementary 5). The composition of plagioclase inclusions and matrix plagioclase is close to pure albite (An0-1) (Fig. 12a; Supplementary 5).

#### Mica

EPMA analyses of biotite (34 points) and muscovite (75 points) and their structural formulae are given in Supplementary 5. In zinnwaldite-muscovite granites, biotite shows high average values of SiO_2_ (42.3 wt%) and Al_2_O_3_ (21.2 wt%), whereas biotite of biotite granites is characterized by the highest values of TiO_2_, FeO^t^, and MgO (3.62, 24.8, 7.46 wt%, on average) (Supplementary 5). Their Mg# (molar Mg/(Mg + Fe) ratio) ranges from 0.20 to 0.38 and 0.33 to 0.38 in zinnwaldite-muscovite granites and biotite granites, respectively (Supplementary 5). In the 10*TiO_2_-(FeO^t^+ MgO)-MgO ternary classification diagram of Nachit et al^80^., the analyzed biotites from biotite granites plot in the primary biotite field, while the biotites of zinnwaldite-muscovite granites plot in the field of secondary biotite and re-equilibrated primary biotite (Fig. 12b). In the Al_2_O_3_ vs. FeO^t^ biotite discrimination diagrams of Abdel-Rahman^81^, all biotites plot in the peraluminous field (Fig. 12c).

According to the ternary classification diagram of Miller et al^82^., the analyzed muscovites of the two granitic suites are classified as both primary and secondary muscovite, fitting to their textural occurrences as described above (Fig. 12d). The primary muscovite has higher contents of TiO_2_ (0.40 to 0.58 wt%), K_2_O (10.32 to 10.76 wt%), but lower Na_2_O (0.17 to 0.30 wt%), Al_2_O_3_ (27.8 to 31.2 wt%), and MgO (0.76 to 2.22 wt%) than secondary muscovite (0.11 to 0.37 wt% TiO_2_, 28.38 to 35.25 wt% Al_2_O_3_, 9.99 to 10.79 wt% K_2_O, 0.08 to 0.48 wt% Na_2_O, and 0.35 to 1.97 wt% MgO). The primary muscovite has TiO_2_ > 0.4 wt%, comparable to muscovite of magmatic origin^83^.

#### Zinnwaldite

The chemical analyses and structural formulae of the zinnwaldite are listed in Supplementary 5. They show a variable chemical composition, with SiO_2_ ranging from 37.7 to 48.8 wt%, Al_2_O_3_ from 16 to 27 wt%, FeO^t^ from 9.3 to 23.5 wt%, and F from 4.41 to 8.05 wt% (Supplementary 5).

#### Zircon

EPMA analyses of zircon (51 points) from both zinnwaldite-muscovite granites and biotite granites indicate a wide variation in its composition and totals (94.5–100.1 wt%) (Supplementary 5), with ZrO_2_ ranging from 58.5 to 64.4 wt%, SiO_2_ from 31 to 34 wt%, and HfO_2_from 1.94 to 6.62 wt% (Supplementary 5). In the evolutionary diagram for zircons from highly evolved rare metal-bearing granites from the CED of Egypt after Abdalla et al^84^., the analyzed zircons plot close to the trend of normal zircon from granitic rocks and between magmatic and metasomatic fractionation trends (Fig. 12e).

#### Columbite

Columbite (72 points) has been analyzed in zinnwaldite-muscovite granite. Its chemical analyses and structural formulae are listed in Supplementary 5. Columbite analyses are plotted together with those of columbite from other localities in the Egyptian Eastern Desert in the Mn# [Mn/(Mn + Fe)] versus Ta# [Ta/(Nb + Ta)] diagram (Fig. 12f). In the quadrilateral diagram, the analyzed columbite mainly plots on the Mn-dominant side of columbite compositions similar to that of the Nuweibi area. All other analyzed columbites occupy the Fe-dominant columbite field, similar to columbite from Abu Rusheid and Mueilha (Fig. 12f). Columbite displays a very wide range of Nb_2_O_5_ (39.1 to 73.5 wt%), Ta_2_O_5_ (4.42 to 43.3 wt%), FeO^t^ (3.17 to 16.0 wt%), and MnO (4.98 to 14.2 wt%) values, reflecting complex zoning patterns.

#### Topaz

Topaz (18 points) has been observed only in zinnwaldite-muscovite granites, and its chemical analyses and structure formulae are listed in Supplementary 5. Topaz has a homogeneous chemical composition, with SiO_2_ ranging from 30.5 to 32.3 wt%, Al_2_O_3_ from 51.7 to 55.1wt%, and F from 14.6 to 16.6 wt% (Supplementary 5).

### Whole-rock geochemistry

The major oxides, trace elements, and CIPW norm data of seventeen representative samples of zinnwaldite-muscovite granites and biotite granites of the Abu Rusheid-Sikait area are listed in Supplementary 6. The studied granites vary in their silica content (69.7 wt% to 82.7 wt%) and differentiation index (D.I = 92.5 to 98.5) (Supplementary 6). Garnet-muscovite granites, zinnwaldite-muscovite granites, and pegmatites have high SiO_2_ (72.2–82.7 wt%), FeO^t^ (0.67–2.83 wt%), moderate Al_2_O_3_ (9.19–13.5 wt%), and Na_2_O+K_2_O (5.94–8.55 wt%) compared to biotite granites (SiO_2_: 69.7–77.2%, FeO^t^ 0.19–0.75 wt%, Al_2_O_3_ 13.5–16.5 wt%, and Na_2_O+K_2_O = 5.93–11.3 wt%) (Supplementary 6, 7 − 1). Garnet-muscovite granites and zinnwaldite-muscovite granites are characterized by high rare-earth element contents (ΣREEs: up to 1007 ppm), as well as high Zr (3800 ppm), Nb (1370 ppm), Ta (216 ppm), Y (693 ppm), Th (503 ppm), U (149 ppm), Hf (207 ppm), and Pb (964 ppm). On the other side, biotite granites show lower ΣREEs (up to 92.1 ppm), and contents of Zr (132 ppm), Nb (162 ppm), Ta (18.8 ppm), Y (55.9 ppm), Th (25.4 ppm), U (9.25 ppm), Hf (6.97 ppm), and Pb (17.3 ppm) (Supplementary 6, 7 − 1). On the other side, pegmatites in the area, which are also highly mineralized, exhibit significantly higher concentrations of several metals compared to the surrounding granites. These pegmatites are particularly enriched in ∑REE (822–1310 ppm), Zr (2865–4477 ppm), Nb (1286–1500 ppm), Y (1145–1436 ppm), Th (413–480 ppm), U (223–411 ppm), Ta (139-145ppm), and Hf (199–242 ppm) (Supplementary 6, 7 − 1).

According to the classification diagram (based on point counting) of Quartz-Alkali feldspar-Plagioclase (QAP) developed by Streckeisen^87^, the studied biotite granites and zinnwaldite-muscovite granites are categorized within the syenogranites field, while garnet-muscovite granites and pegmatites straddle the boundary between syenogranites and alkali feldspar granites (Supplementary 7-2a). Similarly, the classification diagram proposed by Middlemost^88^ places both types of granite within the granite field (Supplementary 7-2b). Analysis through Harker variation diagrams reveals consistent differentiation patterns in the examined granites (Supplementary 7 − 3). As the concentration of SiO_2_ increases, there is a corresponding decrease in the contents of TiO_2_, Al_2_O_3_, MgO, FeO^t^, CaO, MnO, Na_2_O, and K_2_O (Supplementary 7 − 3), due to the fractionation of felspar, providing arguments against albitization as an important alteration process.

**Fig. 13:**
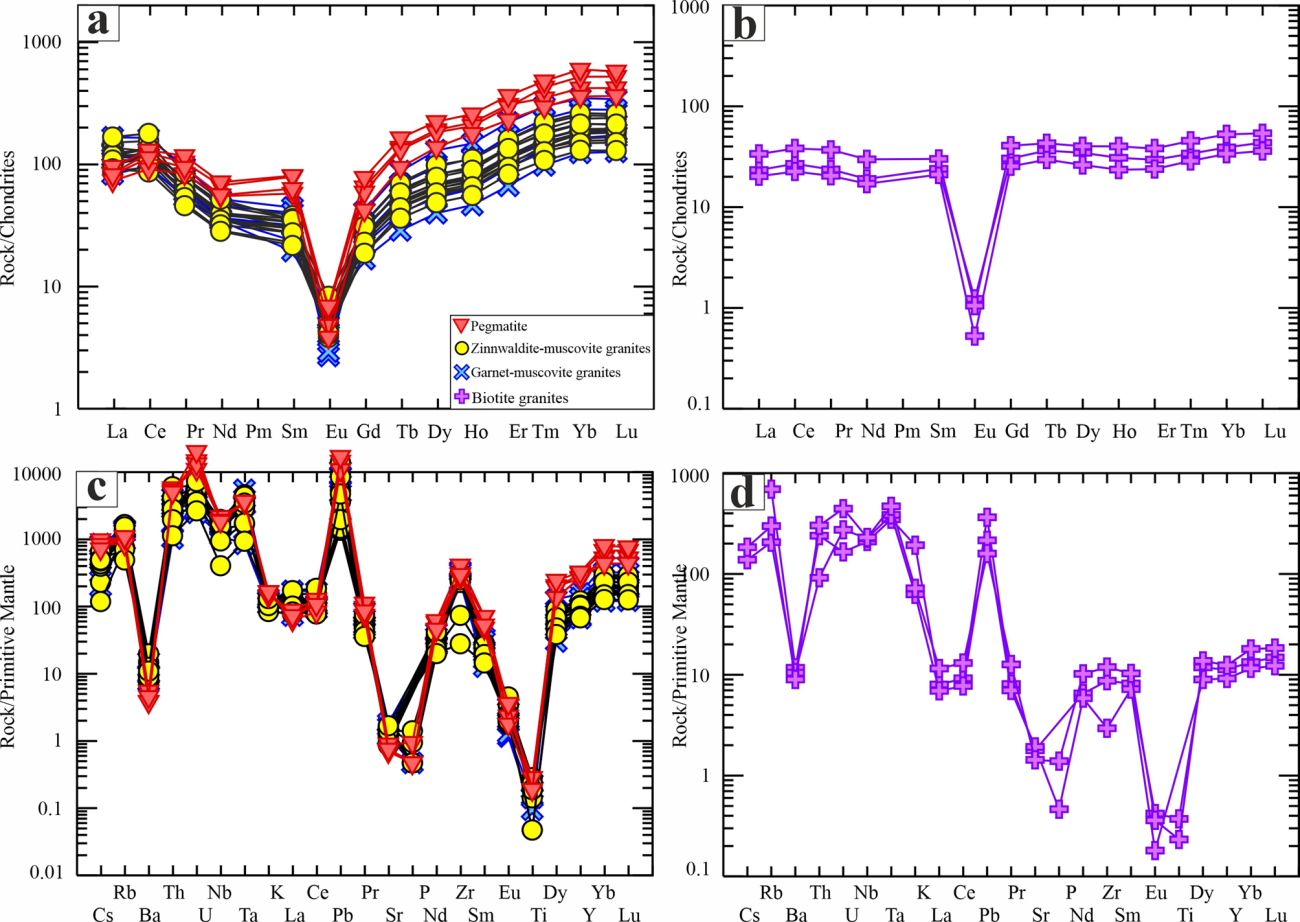
Normalized multi-trace element diagrams for whole-rock chemistry. Chondrite-normalized REE patterns of garnet-muscovite granites, zinnwaldite-muscovite granites, and pegmatites (**a**) and biotite granites (**b**) of the Abu Rusheid-Sikait area. Primitive mantle-normalized trace elements plots of garnet-muscovite granites, zinnwaldite-muscovite granites, and pegmatites (**c**) and biotite granites (**d**). Chondrite values from Sun and McDonough^78^.

The chondrite-normalized rare earth element (REE) patterns, utilizing normalization factors of Sun and McDonough^89^, reveal that the studied granites exhibit enrichment in heavy rare earth elements (HREEs) compared to light rare earth elements (LREEs), alongside pronounced negative Eu anomalies. This is particularly evident in the case of the garnet-muscovite granites, zinnwaldite-muscovite granites, and pegmatites (Eu/Eu* = 0.04–0.24), in agreement with their strongly fractionated composition and enrichment in zircon, xenotime, thorite, monazite, and fluorite. Biotite granites display a flatter pattern with less pronounced negative Eu anomalies (Eu/Eu* = 0.02–0.05) (Fig. 13a, b) (Supplementary 6). Additionally, primitive mantle-normalized trace element diagrams for the studied granites (Fig. 13c, d) indicate negative anomalies for elements such as Ba, Ta, Sr, Pb, Eu, and Ti, with a negative correlation between FeO^t^, Al_2_O_3_, CaO, and SiO_2_ (Supplementary 7 − 3). This suggests that fractional crystallization processes involving plagioclase, zircon, and Fe-Ti oxides have occurred. Furthermore, the studied granites and pegmatites exhibit REE tetrad effects, with TE_1,3_ values ranging from 1.13 to 1.21 in the biotite granites, 1.36 to 1.49 in garnet-muscovite granites, 1.36 to 1.61 in the zinnwaldite-muscovite granites, and 1.47 to 1.61 in pegmatites, indicating the influence of late to post-magmatic fluids during their differentiation process.

The studied rock samples are mainly unaltered, except for one sample of biotite granites (SK15) and three samples of zinnwaldite-muscovite granites (ARS7, ARS15, and ARS22a), which have undergone minor sericitization (Supplementary 6). Their chemical compositions are nearly identical to those of the unaltered samples, aside from minor modifications in certain major oxides and trace elements, i.e. weak increases in SiO_2_, Na_2_O, FeO^t^, MgO, U, Pb, Rb, Ba, Cr, Sc, Zn, Sm, and Ni, decreases in K_2_O and CaO (Supplementary 6, 7 − 3). Based on the LOI values versus mobile elements diagram, the concentrations of K_2_O and CaO contents in the slightly altered samples display a negative correlation with LOI values (Supplementary 7-4a-d). In contrast, Na_2_O, FeO_t_, and MgO significantly increase as LOI rises (Supplementary 7-4e, f). The U-enrichment is related to the ferrugination alteration as it rises with increasing FeO^t^ contents (Supplementary 7 − 5). Moreover, the U makes a positive correlation with some trace elements like Zr, Nb, Ta, Zn, Y, Ce, Pb, and Cu (Supplementary 7 − 6).

## Discussion

### Machine learning-based lithological mapping

This study establishes the application of pixel-based machine learning algorithms like Random Forest (RF) and Support Vector Machine (SVM) to hyperspectral PRISMA data for lithological discrimination in the Wadi Abu-Rusheid-Sikait granitoid complex. The results demonstrate the efficacy of these algorithms in accurately mapping lithological units, validated through field observations, petrographic investigations, and geochemical analyses (Figs. 2, 3, 4, 5, 6 and 7; Supplementary 3–7). Both classifiers achieved high overall accuracies (RF: 88.13%, SVM: 89.02%) when applied to Minimum Noise Fraction (MNF)-transformed data, highlighting the critical role of preprocessing in enhancing hyperspectral data utility (Fig. 5c-f; Supplementary 3 − 1). Particularly, the MNF transformation consistently outperformed raw PRISMA bands, improving noise reduction and feature extraction^5,19,20^.

The RF classifier exhibited robust performance across various lithologies, with overall accuracies of 86.28% (original bands) and 88.13% (MNF-transformed data). Specific units, such as ophiolitic mélange, ophiolitic metagabbros, and alkali feldspar granites, achieved excellent classification accuracy (> 88%) in both datasets due to their unique mineral assemblages producing diagnostic absorption features (Fig. 5c, d). This highlights RF’s ability to handle complex spectral variations inherent to these units. Units with subtle mineralogical differences, such as zinnwaldite-muscovite granites and garnet-muscovite granites (Figs. 9, 10 and 11; Supplementary 6), showed marked improvement in the MNF dataset, with F1-scores rising to 91.25–92.75%, suggesting that noise reduction enhances discrimination of spectrally similar lithologies. However, classification errors were most pronounced for gneissic rocks (F1-score: 78.37%) and biotite granites (79.94%), attributed to their heterogeneous composition, variable metamorphic textures, and spectral overlap with other units, as evidenced by confusion matrix analysis showing misclassification primarily with tonalites-granodiorites and alkali feldspar granites due to similar feldspar-quartz assemblages (Fig. 5c, d; Supplementary 3 − 1). The RF algorithm’s ensemble approach, utilizing multiple decision trees with random feature selection, proved particularly effective for handling the high-dimensional hyperspectral data and managing class imbalances, as demonstrated by the relatively consistent performance across units with varying pixel populations (from 943 pixels for zinnwaldite-muscovite granites to 6,651 for metagabbros), though the lower sample size for rare lithologies like zinnwaldite-muscovite granites may have contributed to reduced recall rates (90.78%) despite high precision (91.83%), highlighting the importance of balanced training datasets in complex geological terranes with variable exposure and accessibility constraints (Supplementary 3 − 1).

Similarly, the SVM classifier (using an RBF kernel) achieved its highest accuracy (89.02%) with MNF-transformed data, outperforming raw bands (84.25%) (Fig. 5e, f; Supplementary 3 − 1). The MNF enhancement significantly improved F1-scores across all units (86.63–93.19%), except for gneisses, which still maintained a respectable F1-score of 81.83%. This divergence likely reflects the heterogeneous composition and textural complexity of gneisses, which challenge spectral separability even after preprocessing (Fig. 2a). The high kappa coefficient (87.41%) and mean F1-score (88.61%) for SVM further validate its reliability in lithological mapping, particularly for units with distinct spectral signatures like Li-rich pegmatitic granites. In the PRISMA data, the success of both classifiers in discriminating lithologies such as ophiolitic metagabbros and alkali feldspar granites (> 93% F1-score) can be attributed to their unique mineral assemblages, which produce diagnostic spectral features in PRISMA data. Conversely, classes like zinnwaldite-muscovite granites and Li-rich pegmatitic granites exhibited moderate recall (61.5–81.58%), likely due to their mineralogical overlap with other granitoids (Figs. 9, 10 and 11; Supplementary 5, 6) and limited training samples (Supplementary 3 − 1). These findings highlight the effectiveness of data processing, such as MNF transformation, in enhancing the performance of machine learning algorithms for lithological mapping^5,19,20^.

Our model’s performance is directly governed by the interplay between the spectral distinctiveness of specific mineral assemblages and the 30 m spatial resolution of the PRISMA sensor (the “mixed-pixel” effect). The high accuracy (> 88%) achieved for Ophiolitic Mélange and Metagabbros is attributed to their strong, broad absorption features in the Mg-OH/Fe-OH region (2300–2350 nm) and low albedo, which remain spectrally distinct from felsic units even within mixed pixels. Conversely, the persistent misclassification of Gneisses arises because their compositional banding (mm- to cm-scale) is homogenized at the 30 m pixel scale, creating a “spectral average” that mimics the bulk composition of tonalites-granodiorites. Similarly, the confusion between biotite and zinnwaldite granites (Supplementary 3 − 2) reflects a fundamental detection limit: both rocks are rich in quartz and feldspar (spectrally featureless in the SWIR), and the diagnostic mica species constitute < 10 vol% for biotite granites and.

≤ 15 vol% for zinnwaldite-muscovite granites, a signal easily diluted or obscured in a 30 m pixel, necessitating the use of MNF transformation to isolate these subtle spectral variances.

Overall, the results highlight the potential of integrating advanced remote sensing techniques with machine learning to successfully generate a precise lithological map and reveal a previously unidentified distribution of granitic rocks in the Abu Rusheid-Sikait area (Figs. 1 and 5). This detailed map is particularly significant, as these granitic rocks are highly enriched with strategically valuable rare metals, such as REEs, Zr, Nb, Y, Th, and U (Supplementary 6).

### Geochemical characteristics and tectonic setting

**Fig. 14:**
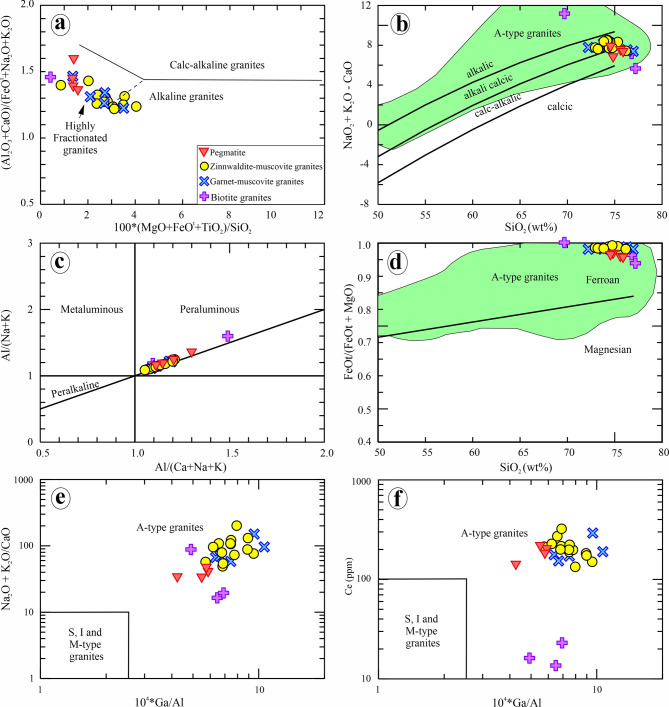
(a) Major element classification diagram (SiO_2_ > 68%), showing the fields of alkaline, calc-alkaline, and highly fractionated calc-alkaline rocks^90^. (b) Chemical classification diagram using SiO_2_ versus Na_2_O + K_2_O − CaO^91^. (c) A/NK (molar Al_2_O_3_/Na_2_O + K_2_O) versus A/CNK (molar Al_2_O_3_/CaO + Na_2_O + K_2_O)^92^. (d) SiO_2_ versus FeOt/FeOt + MgO binary diagram showing that the Abu Rusheid-Sikait granitoids are ferroan^86^. 104*Ga/Al against Na_2_O+K_2_O/CaO (e) and Ce (f) for distinguishing between I, S, M, and A-type granites^93^.

In this study, a variety of geochemical classifications and discrimination diagrams for granitic rocks are used to critically evaluate the studied granites (Fig. 14). The Abu Rusheid-Sikait granites and pegmatites can be characterized as highly fractionated calc-alkaline magmatic suites, in accordance with the major element classification diagram of Sylvester^90^ (Fig. 14a). Generally, these granites exhibit elevated alkali contents (Na_2_O + K_2_O = 5.93 to 11.2 wt%; Supplementary 6) and predominantly fall within the calc-alkalic to alkali-calcic fields of Frost et al^91^. (Fig. 14b). They exhibit peraluminous nature (molar A/CNK: (Al_2_O_3_/CaO + Na_2_O + K_2_O) = 1.05–1.5) (Fig. 14c), which is supported by the normative corundum (0.68–4.65; Supplementary 6), as well as the identification of muscovite and garnet within the thin sections (Figs. 9, 10 and 11). Additionally, they are considered ferroan A-type granites (Fig. 14d) as indicated by the FeOt/(FeO + MgO) vs. SiO_2_diagram after Frost et al^91^..

**Fig. 15:**
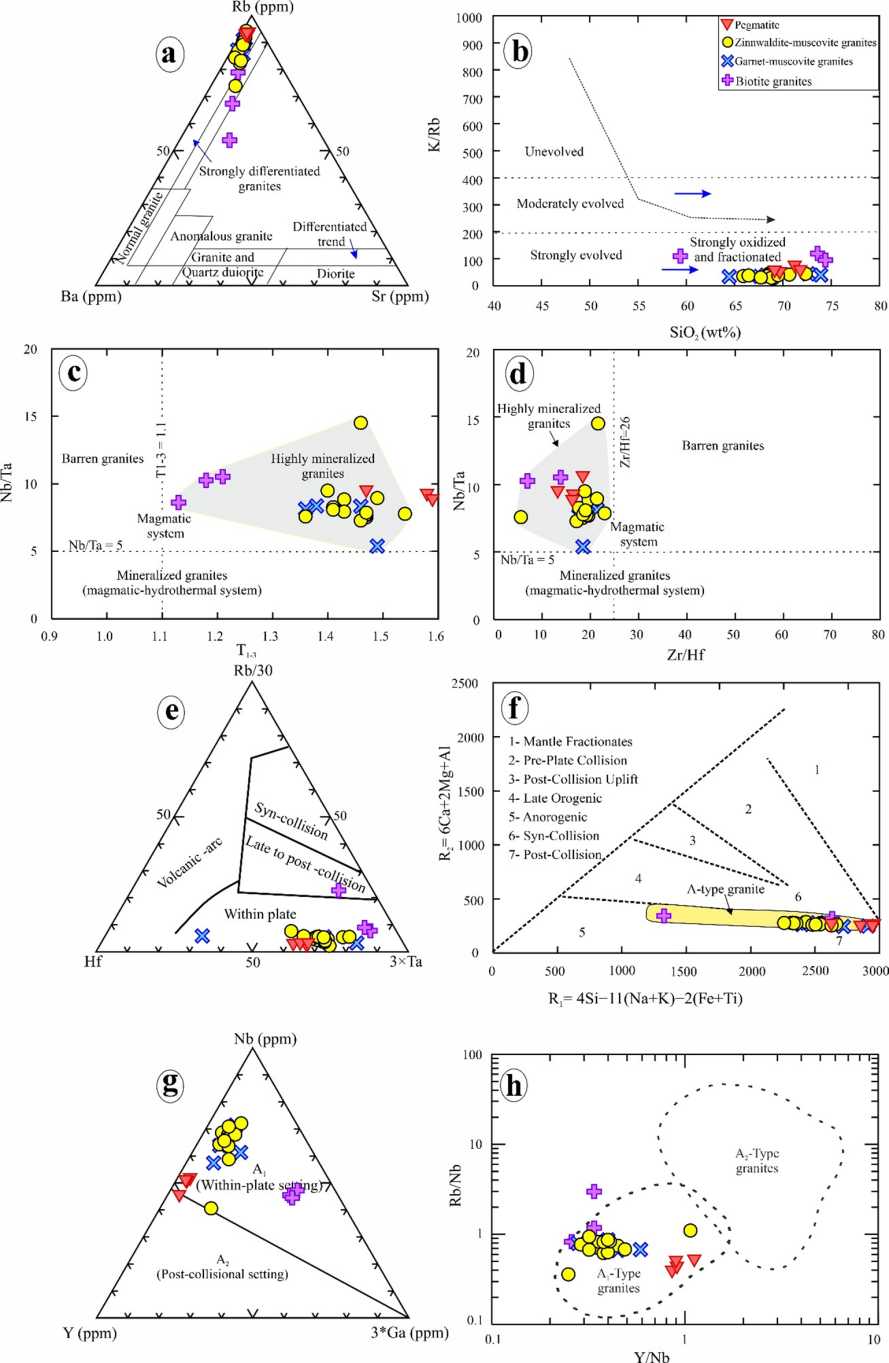
a) Plot of the Abu Rusheid-Sikait samples on Sr, Rb, and Ba triangular diagram^96^. (b) Plot of K/Rb vs. SiO_2_^97^, showing that the Abu Rusheid-Sikait granites are highly evolved, oxidized, and fractionated. (c) Hf-Rb/30–3*Ta ternary diagram (after Harris et al.^95^. Binary relations between Nb/Ta Vs. TE1-3 (d) and Nb/Ta vs. Zr/Hf (e) for Abu Rusheid-Sikait granitoids. Horizontal dashed lines at Na/Ta = 5 mark the transition from a pure magmatic (geochemically specialized) and magmatic-hydrothermal (mineralized) systems^98^, while the vertical dashed lines at TE_1−3_=1.1 and Zr/Hf = 26 separate the barren and mineralized rocks. (f) R1-R2 multicationic diagram^99^. Abbreviations: R1 = 4Si-11(Na + K)−2(Fe + Ti); R2 = 6Ca + 2 Mg + Al. (g) Y-Nb-Ga*3 ternary diagram and Rb/Nb versus Y/Nb binary diagram (h) of Eby^100^ to distinguish between A1 and A2 granitoids; A1 is anorogenic A-type granite with mantle signature, whereas A2 is post-collisional A-type granite with crustal source.

The studied granitoids show enrichment in FeOt/MgO, Ga/Al, HFSE (Zr, Hf, Y, and Nb), alongside a significant depletion in CaO, MgO, and P_2_O_5_ (Supplementary 6). These chemical aspects characterize post-collisional A-type granites^1,2,5,6,8,9,16,55,93,94^. In the binary diagrams of (Na_2_O +K_2_O) versus 10^4^×Ga/Al and of Ce versus 10^4^×Ga/Al of Whalen et al^93^., the studied granitoids are classified as A-type granites (Fig. 14e, f). They are characterized by a depletion in compatible trace elements such as Ba, Sr, Ni, and V, while exhibiting an enrichment in incompatible trace elements like Nb, Ta, Y, Th, and U (Supplementary 6). The highly evolved nature of these granitoids and pegmatites was confirmed by the Rb–Ba–Sr ratios and SiO_2_ vs. K/Rb diagram (Fig. 15a, b). Moreover, the studied granites are highly fractionated regarding their (Gd/Yb)n ratios of 0.74 to 0.76 in biotite granites and of 0.05 to 0.11 in highly mineralized granites and pegmatites (Supplementary 6). Therefore, they display the chemical characteristics of highly mineralized granites on the Nb/Ta versus T_1−3_ and Zr/Hf diagrams (Fig. 15c, d).

In this work, the tectonic setting of the granitic rocks and pegmatites was elucidated using multiple tectonic discrimination diagrams (Fig. 15e-h). In the Rb/30-Hf-3×Ta ternary diagram of Harris et al^95^., the Abu Rusheid-Sikait granitoids and pegmatites are situated within the field of within-plate granites (Fig. 15e). Moreover, in the modified multicationic diagram R1-R2 (Fig. 15f), they lie dominantly in the field of post-collisional granitoids^100^. delineates A-type granites into two classifications, namely A1 and A2, wherein the A1 classification is characterized by mantle-derived origins and is emplaced in an anorogenic context. In contrast, the A2 classification is constituted by crustal-derived magmas that are indicative of a post-orogenic environment. Our granites fall within the A1 classification in the Nb-Y-Ga×3 and Y/Nb versus Rb/Nb ternary diagrams (Fig. 15g, h), suggesting a within-plate tectonic setting.

In the following paragraphs, the geodynamic implications of the new data for the Abu Rusheid-Sikait granites will be discussed. Field relations demonstrate that the investigated granites form small, individual plutonic bodies which postdate regional subduction-related magmatism and are cross-cut by within-plate alkali feldspar granites of Gabal Zabara to the north (Figs. 1c and 2). Petrographic investigations indicate sub-solidus deformation, marked by undulose and chessboard extinction in quartz, albite deformation twins, and muscovite kinking, possibly associated with reactivation of the Nugrus shear zone of the Najd fault system, as suggested by (Figs. 9 and 10)^61,95^. Geochemically, the Abu Rusheid-Sikait garnet-muscovite granites, zinnwaldite-muscovite granites, and associated pegmatites have similar geochemical properties (Figs. 13, 14 and 15; Supplementary 6, 7), indicating a common source. All the former granites represent highly evolved and strongly fractionated peraluminous A-type granites generated in a post-collisional within-plate environment similar to other A-type granites within ANS^1,2,5,6,8,9,13,15,60^.

Elsagheer et al^60^. proposed a geotectonic model for the evolution of these A-type granites and associated pegmatites (supplementary 7 − 6). According to these authors, structural and geochemical data confirm that regional extension and shear-zone controlled hydrothermal fluid flow focused rare-metal mineralization. Structural controls on rare-metal mineralization at Abu Rusheid–Sikait are clearly demonstrated by the spatial association of mineralized zones with prominent NW–SE, N–S, and NE–SW lineament sets mapped by ALOS PALSAR and Sentinel-1 radar data (Fig. 6a, b). Field observations reveal uranium-rich halos marked by yellow kasolite staining concentrated at pegmatite–granite contacts and shear-zone intersections (Figs. 2e and 3j). Ferruginous (hematite-goethite) alteration aligned along these structures, confirming that structural lineaments were the primary controls on fluid ingress and secondary minerals deposition (Figs. 2d and f, 3 and 5h). Samples affected by ferrugination display elevated FeO^t^ and associated uranium enrichment, indicating adsorption of U^6+^ onto iron oxide surfaces (Supplementary 7 − 2). REE tetrad effects (TE_1,3_ = 1.13–1.61) and negative Eu anomalies (Eu/Eu* = 0.04–0.24) confirm fluid-melt interaction during fractionation and post-magmatic alteration (Fig. 13a, b; Supplementary 6). Hydrothermal minerals like kasolite, fluorite, galena, and topaz provide mineralogical resistance to element remobilization (Figs. 10b, e and h and 11g). The combined structural, mineralogical, and geochemical evidence thus demonstrates that while extreme magmatic fractionation of A-type granites generated primary rare-metal enrichment, subsequent structurally controlled hydrothermal overprint focused and enhanced these metals into economically significant concentrations along the Najd fault and Nugrus shear zones.

### Mineralization and alteration

The investigated granitoids and pegmatites exhibit significant magmatic fractionation, evidenced by elevated Rb (> 200 ppm), low K/Rb (< 200), and depleted Ba (< 200 ppm) and Sr (< 80 ppm) concentrations (Supplementary 6). These rocks are classified as low-phosphorus mineralized granites, which are characterized by high silica (SiO₂ >73 wt%), low alumina (Al₂O₃ <14.5 wt%), and phosphorus (P₂O₅ <0.1 wt%) content^5,13^. The origin of rare metal mineralization in these granites is still a subject of debate. Different scenarios of rare metals-enrichment of the granites are considered, which include magmatic enrichment^2,101,102^, enrichment during hydrothermal alteration (kaolinization, albitization, silicification, chloritization, and sericitization^12^, and a combination of igneous and hydrothermal enrichment processes^1,4–6,8,9,59,100^. Petrographic and mineral chemical analyses confirm a magmatic origin of the main minerals, albite and K-feldspar, as well as of part of the rare metals-bearing phases, such as zircon, columbite, thorite, xenotime, monazite and garnet (Figs. 9, 10, 11 and 12). Magmatic zinnwaldite occurs as large to medium subhedral crystals and corroded crystals containing inclusions of albite (Figs. 9i-o and 10f). Garnet forms euhedral to subhedral crystals containing quartz and zircon inclusions and displaying straight grain boundaries with magmatic phases, reflecting its magmatic origin [Figs. 1, 8 and 9d and e]. On the other side, the interstitial crystallization of topaz, observed as texturally late-stage infill between muscovite and albite crystals, points to the infiltration of fluorine-rich hydrothermal fluids during the final stages of magmatic cooling (Fig. 10e)^103^. Additionally, some feldspar, muscovite, and biotite crystals were modified later by hydrothermal solution and altered to sericite, chlorite, kaolinite, and Fe oxide (Figs. 9, 10 and 11). Based on the textural relationships and compositions of the rare metals-bearing minerals in the studied samples, the rare metals-enrichment resulted from different processes, which will be discussed in detail in the following sections.

#### REE, U, and Th enrichment

In the Abu Rusheid-Sikait granitoids and pegmatites, the main REE carriers are zircon, columbite, phosphate minerals (xenotime, monazite), and radioactive minerals (thorite, uranothorite, and kasolite) (Figs. 9, 10 and 11; Supplementary 5). Zircon is the predominant accessory phase in the studied granites, with contents of up to 3 vol% in zinnwaldite-muscovite granites (Figs. 9, 10 and 11). It includes medium-grained subhedral to euhedral prismatic as well as wedge-shaped crystals, both showing an oscillatory magmatic zonation (Figs. 9, 10 and 11), indicating its magmatic formation. Its magmatic origin is confirmed by Si/Zr ratios (1.08–1.10, Supplementary 5). Notably, the composition of the analyzed zircons ranges from Zr0.97Hf0.06SiO4 to Zr0.89Hf0.02SiO4, similar to magmatic zircon in other peraluminous Li-rich granites in Egypt (Supplementary 5)^78^.

The magmatic zircon crystals are rich in inclusions, like columbite and thorite (Fig. 11a). Like zircons from other localities in Egypt, those of Abu Rusheid-Sikait plot close to granitic zircons and in between magmatic and metasomatic zircon trends in the HfO_2_ versus ZrO_2_diagram after Abdalla et al^84^. (Fig. 12e). Zircon typically displays normal zoning, characterized by rims that consistently show higher Hf concentrations compared to the cores. Notably, zircon is associated with topaz, tantalite, zinnwaldite, and lithium-rich white mica in the zinnwaldite-muscovite granites (Figs. 9, 10 and 11). The textural occurrence and composition of columbite support its magmatic origin, since most of the columbites are chemically homogeneous and frequently occur as inclusions in quartz, K-feldspar, muscovite, and zircon (Figs. 9e and 11a, b and d). Some columbite crystals exhibit a normal zoning pattern, characterized by increasing Ta_2_O_5_ towards the rim (Supplementary 5). In zinnwaldite-muscovite granites, columbite crystals are associated with cassiterite and occur as inclusions in quartz (Figs. 9e and 11d). This mineral association, as well as the high Nb/Ta ratios (7–28-14.5.5; Supplementary 6) of the zinnwaldite-muscovite granites, suggest that the predominance of Nb over Ta is due to igneous fractionation processes^84^. EDS analysis of cassiterite indicates that it has Ta_2_O_5_ over Nb_2_O_5_ (Supplementary 4), similar to Li-rich albite granites of the Igla and Abu Dabbab areas in Egypt^84,104^. It is suggested that cassiterite crystallized simultaneously with or formed early after columbite in the magmatic stage. Likewise, thorite and U-rich thorite (uranothorite) occur as inclusions in primary phases like quartz and zircon (Figs. 9e and 11a and c), indicating their magmatic origin. Xenotime (YPO_4_) forms subhedral crystals in the interstitial spaces between quartz and K-feldspar in the studied zinnwaldite-muscovite granites and as inclusions in zircon and columbite crystals. Monazite [LREE, Th(PO_4_)] is a common accessory mineral in the studied granites, particularly zinnwaldite-muscovite granites. They are mainly euhedral to subhedral crystals with pleochroic halos in the magmatic muscovite, zircon, and columbite minerals, reflecting their magmatic origin (Figs. 10f and 11e). The studied monazite crystals have high LREE, P_2_O_5_, and Th contents (Supplementary 4). The textural relationship of xenotime and monazite indicates their magmatic origin^104^.

Kasolite (Pb(UO_2_)SiO_4_.H_2_O), which occurs together with fluorite in part of the samples, formed as a secondary uranium mineral replacing a metamict primary radioactive phase (likely zircon) (Fig. 10b), indicating that both kasolite and fluorite formed during hydrothermal alteration. In the field, enrichment in kasolite occurs close to the fractures in granites and along the Nugrus shear zone (Figs. 2e and 11k). Microscopically, abundance of kasolite is observed to be associated with ferrugination alteration (Fig. 10d, h). In the studied granites, uranium shows a positive correlation with FeO^t^, suggesting uranium adsorption on iron oxides or hydroxides (hematite/goethite) due to interaction with U-rich hydrothermal solutions (Supplementary 7 − 5). Geochemical analyses reveal strong positive correlations between uranium and high-field-strength elements (Zr, Nb, Ta, Hf) and rare earth elements (Ce, Y) (Supplementary 7 − 6), suggesting that enrichment is caused primarily by magma fractionation and only redistributed and locally concentrated during secondary alteration^54^.

 In summary, the petrography and mineral chemistry data indicate that the REE, U, and Th mineralizations of the investigated Abu Rusheid-Sikait granitoids mainly result from magmatic fractionation processes and are concentrated in magmatic phases. This interpretation also fits the observed geochemical characteristics of the granitoids, i.e., (1) their peraluminous nature (Fig. 17c; Supplementary 6); (2) the alkali-rich composition (Table 2; Supplementary 6); (3) their volatile-rich (e.g., H_2_O, F) nature with abundant primary mica (muscovite and biotite) (Figs. 9, 10 and 11; Supplementary 5), and (4) their A-type characteristics^85,105^. Secondary alteration, mainly marked by the frequent formation of Fe oxides and hydroxides together with fluorite and kasolite, is associated with U redistribution and local concentration along faults and fractures.

Notably, the zinnwaldite-muscovite granites, garnet-muscovite granites and pegmatites, which are strongly fractionated, demonstrate significantly higher concentrations of REEs (up to 1310 ppm), Zr (4477 ppm), Nb (1500 ppm), Ta (182 ppm), Y (1436 ppm), Th (503 ppm), and U (411 ppm), compared to biotite granites (Fig. 15; Supplementary 6), confirming that they are more differentiated. LREE enrichment is mainly related to the presence of magmatic monazite, while the HREE enrichment is due to zircon, xenotime, and fluorite. This indicates a marked enrichment of these metals during the magmatic crystallization of rare metals-bearing minerals, such as zircon, xenotime, monazite, thorite, and uranothorite. The effect of the post-magmatic stage, with the REE being remobilized by hydrothermal fluids, is restricted to the formation of minor kasolite and fluorite.

#### Sulfide mineralization

Sulfide minerals are rare in the studied granites and are mainly represented by galena in the zinnwaldite-muscovite granites (Fig. 11g). The textural occurrence of galena points to its secondary formation during hydrothermal alteration. In the magmatic stage, plagioclase, mica, and other related high-field-strength elements, including accessory monazite, xenotime, zircon, and columbite-group oxides, are the principal carriers of Pb. The leaching of Pb from their original sites in the lattices of these rock-forming minerals during alteration is controlled by the presence of F and Cl species in the fluids^105^.

#### Hydrothermal alteration

In this study, the Analytical Spectral Device (ASD) has been used to obtain the reflectance spectra and alteration minerals from fourteen rock samples, which represent a variety of zinnwaldite-muscovite granites and biotite granites from the Abu Rusheid-Sikait area (Fig. 7; Supplementary 3 − 2). Utilizing the ASD-TerraSpec Halo near-infrared (NIR) hyperspectral instrument, capable of measuring reflectance spectra over a spectral range of 400–2500 nm, our field samples were analyzed to extract the implemented spectra of biotite granites and zinnwaldite-muscovite granites, thereby identifying their spectral signatures along with those of typical alteration minerals (Fig. 7; Supplementary 3 − 2). The results of the mineral mapping in the Abu Rusheid-Sikait area, utilizing the hyperspectral PRISMA (Fig. 6) dataset, provide a comprehensive understanding of the mineralization processes and their spatial distribution in the area. As mentioned in the previous paragraph for ASD measurements, the study employed eight mineral indices to detect and map distinct alteration zones, each associated with specific mineral assemblages and spectral characteristics (Fig. 6). The following sections will discuss these zones in detail.

##### Phyllic Al-OH-bearing alteration

Phyllic alteration is generally regarded as a very important indicator for mineralization, such as REE, gold, and porphyry copper deposits^4,5,9,10^. It is characterized by the increasing abundance of sheet minerals such as sericite, illite, and smectite. These minerals exhibit a strong Al-OH absorption feature, typically centered between 2185 and 2225 nm, which corresponds to PRISMA band 194 (Supplementary 4–4, 4–5). The zones identified by the PRISMA indices (b201/b194) and (b189 + b201)/(b194), which are designed to detect muscovite and other phyllic minerals. Microscopically, plagioclase, K-feldspar, and primary muscovite are altered to secondary white mica, reflecting phyllic alteration (Fig. 9a, c, d). Mineral chemical analysis further validated this, distinguishing the fine-grained, secondary muscovite (with specific Al₂O₃ and Na₂O contents, Supplementary 5) from primary magmatic crystals. Notably, the most extensive alteration is observed along the margins of the granitic plutons, particularly the garnet-muscovite granites, Li-pegmatite granites, alkali feldspar granites, and tonalites-granodiorites (Figs. 2 and 6a and b).

##### Argillic alteration

**Fig. 16:**
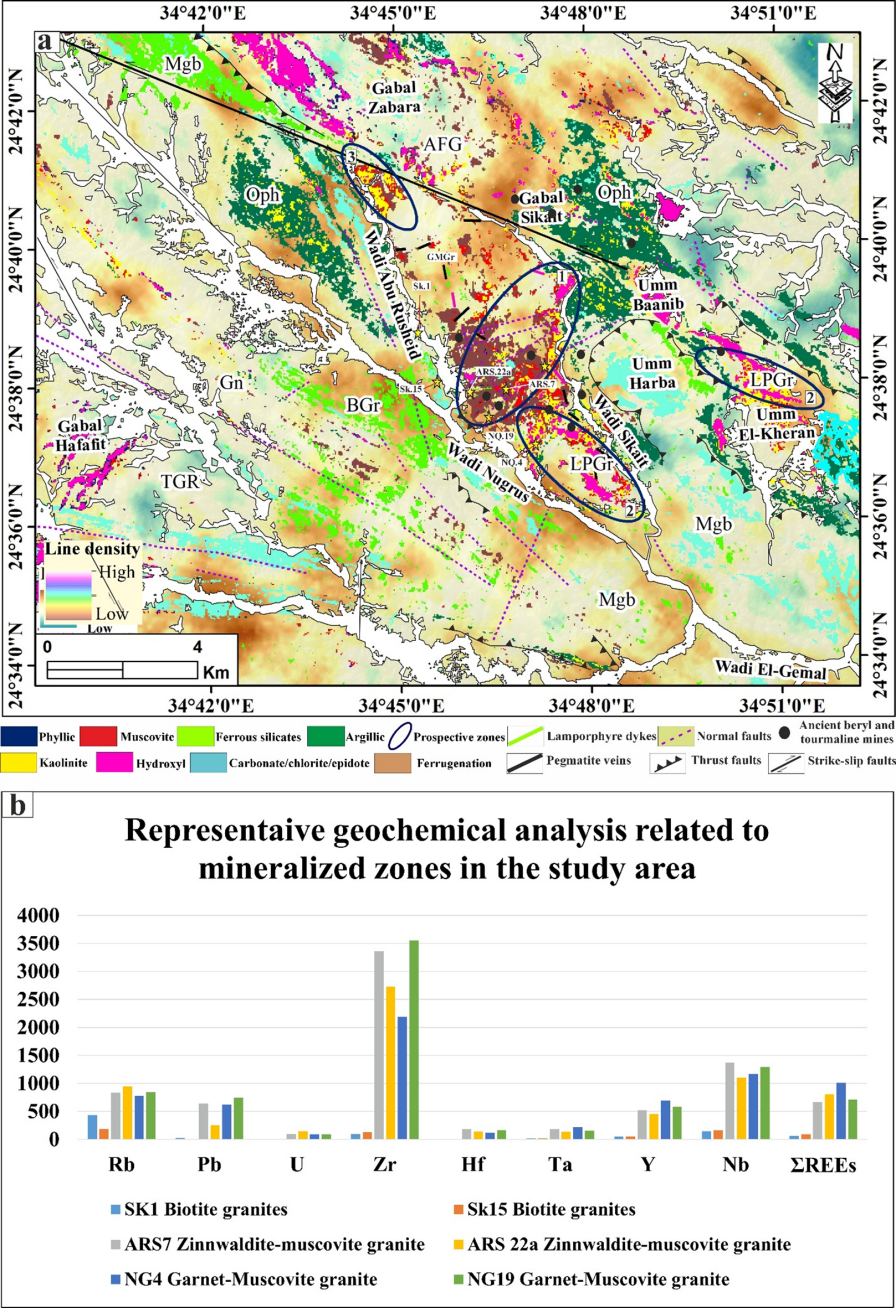
**(a)** Integrated prospectivity map demonstrating the synergistic application of machine learning algorithms applied to PRISMA hyperspectral, alteration mapping, radar-derived structural analysis, and ground-truth validation through field work, petrography, and geochemistry. The map successfully delineates three high-priority exploration zones (1–3) based on convergence of: (1) validated alteration assemblages (phyllic, argillic, propylitic, ferrugination), (2) structural complexity indicated by lineament density, and (3) highly evolved granite units (GMGr, ZMGr, LPGr) confirmed through petrographic investigation and geochemical analysis. (**b**) Representative geochemical analysis related to the mineralized and less mineralized zones. This integrated approach demonstrates the higher targeting ability achieved through combining automated remote sensing analysis with traditional geological methods, providing quantified exploration priorities for rare-metal mineralization in the Abu Rusheid–Sikait area. Created by ArcGIS Desktop 10.8; https://www.esri.com/en-us/arcgis/products/arcgis-desktop/overview.

Argillic alteration zones are generally marked by minerals such as kaolinite, alunite, and pyrophyllite, which were identified using PRISMA data due distinct absorption feature at 2165 nm, corresponding to PRISMA band 189 (Fig. 6c, d), with additional absorption features at 1450 nm and 1950 nm indicating OH-bearing and clay minerals (e.g., kaolinite, montmorillonite) (Table 2; Supplementary 4–4, 4–5). The mapping of these zones is significant because the argillic alteration is often associated with the leaching of primary minerals and the redistribution of metals, which can lead to the enrichment of rare metals^5,9,10^. In the study area, argillitic alteration, indicated by PRISMA data, is mainly observed in those granitic rocks, which are characterized by strong fracturing and jointing (Fig. 2c, d, f), which likely facilitates fluid transport^5,9,10^. This is further evidenced by field observation, silica enrichment, ASD measurements, and the ASTER spectral library (Figs. 2d, 10i and 16d; Supplementary 6).

##### Propylitic alteration

Propylitic alteration zones, defined by assemblages of epidote, chlorite, and carbonate minerals, were delineated using a PRISMA-derived spectral index (Fig. 6g). These minerals exhibit diagnostic absorption features in the 2330 nm region (PRISMA band 210), attributed to Fe/Mg-OH bonds in chlorite and epidote, and CO_3_ vibrational modes in carbonates (Fig. 6c, g). Petrographically, chlorite, formed through hydrothermal replacement of magmatic ferromagnesian minerals (e.g., biotite; Fig. 10c), serves as a critical indicator of post-magmatic fluid activity. Carbonate minerals (calcite, dolomite) further contribute to these alteration signatures, with distinct CO_3_ absorption features observed between 2110 and 2335 nm validated by field ASD spectroscopy (Supplementary 3–4, 3–5). This spectral behavior was exploited through PRISMA indices (b201 + b219)/(b210), which contrast carbonate reflectance peaks (2010 and 2190 nm) against absorption troughs (2100 nm), enabling precise mapping of carbonate-rich zones. The integration of mineralogical and spectral data underscores the efficacy of PRISMA in resolving complex alteration patterns linked to hydrothermal fluid pathways.

##### Ferrugination

Ferrugination, in the granitic rock alteration, refers to the process where iron-bearing minerals are oxidized and/or new iron-rich minerals are formed, often resulting in a reddish or brownish coloration (Fig. 2d, f). This alteration is typically driven by hydrothermal or weathering processes. Ferrugination often involves the oxidation of ferrous iron (Fe^2+^) in minerals like biotite and hornblende to ferric iron (Fe^3+^), which then forms iron oxides (like hematite or goethite) or hydroxides. Whole-rock geochemistry shows a strong positive correlation between FeO^t^ and U content in these zones (Table 2; Supplementary 6, 7 − 5, 7 − 6). Ferrugination, characterized by the introduction or alteration of iron-bearing minerals, is frequently associated with uranium and rare earth element (REE) mineralization due to the chemical interactions during hydrothermal alteration. These interactions, often driven by fluids, can cause the mobilization and deposition of uranium and REEs, resulting in their concentration in specific locations, sometimes alongside iron oxides (Fig. 16; Supplementary 6, 7 − 2, 7 − 3).

Hematite and goethite, iron-rich minerals, display strong to moderate absorption features in the visible and near-infrared (VNIR) wavelength region of ca.700–900 nm (Supplementary 4–4, 4–5). These features are consistent with the PRISMA mineral index (b189/b52), which detects varying degrees of ferrugination within the studied granitoids (Fig. 6h). In the field, secondary hematite is found mainly at the surface of the exposed granites, particularly in zinnwaldite-muscovite granites, garnet-muscovite granites, and pegmatites, and is associated with kaolinite and rare metal-bearing minerals, especially kasolite (Fig. 2d, f). Microscopically, ferrugination alteration is associated with secondary uranium mineral (kasolite) (Fig. 10h). Geochemically, the samples affected by ferruginous alteration show significantly higher Fe oxide contents in comparison to the fresh (unaltered) granites (Supplementary 6, 7 − 5). As a result, they might play a significant role in the redistribution and concentration of U in the area. The observed close correlation of Fe-and U-enrichment (Supplementary 7 − 5) is likely related to the high ability of iron oxides for adsorption of radioactive elements from hydrothermal solutions^59^. Uranium is often mobilized in its soluble + 6 oxidation state (U^6+^) in oxidizing fluids. When these fluids encounter reducing conditions (e.g., due to the presence of sulfide minerals. Like galena (Fig. 11g), uranium can be reduced to its + 4 oxidation state (U^4+^) and precipitate as kasolite (Fig. 10b, h), uraninite, or other uranium minerals.

Structural elements, particularly those trending NW-SE, NE-SW, and N-S, identified through ALOS PALSAR and Sentinel-1 automatic extraction (Fig. 8), may play a crucial role in controlling the enrichment of rare metals in the Abu Rusheid–Sikait area. These lineaments could serve as primary conduits for hydrothermal fluids, which overprinted the garnet-muscovite granites, zinnwaldite-muscovite granite, and associated pegmatites. The lineaments align with the Nugrus shear zone within the Najd fault system (Fig. 1), linking the area to regional post-collisional extension in the ANS. For the assumed fault structures in the study area, our validation relies on: (1) field data: during our field work, we analyzed the orientation of fracture sets in zinnwaldite-muscovite granites, garnet-muscovite granites, and associated pegmatites which host mineralization (shown in Fig. 2a and d-g). Those mineralized zones coincide with the dominant NW-SE, NE-SW, and N-S trends identified by the ALOS PALSAR/Sentinel-1 extraction; (2) Petrographic evidence: a structural control on secondary alteration and formation of kasolite, hematite, goethite, fluorite, and galenite, among others, is further evidenced by the microscopic studies. Thin sections from “high lineament density” zones (e.g., the zinnwaldite-muscovite granites) exhibit shear-related microstructures, such as S-C fabrics and undulose extinction of quartz (Figs. 9d and 10j-i). In hand samples, a well-developed shearing is defined by aligned mica flakes (Fig. 3a). These observations confirm that the lineaments mapped by satellite data correspond to major brittle-ductile deformation zones (related to the Nugrus Shear Zone) that likely served as pathways for mineralizing fluids^13,61,62^. For structural controls on mineralization our field evidence, imply concentration of late-magmatic fluids in the marginal zones of the highly fractionated zinnwaldite granite pluton or wall rock-magma interaction/alteration (Figs. 2c-d and 3) and secondary F, U, and Pb mineralization (kasolite, galena, fluorite, topaz) in the sheared marginal zones of the granite plutons (Figs. 2e and 3k), confirms that these fluids were channeled through these structural networks, leading to a certain remobilization and potentially also concentration of rare metals like Pb, U. The fact that these secondary rare metals-bearing minerals are mainly restricted to the alteration zones in the strongly fractionated zinnwaldite-bearing granites and pegmatites and do not occur in the other granites provides strong evidence that remobilization of rare metals is locally restricted to units which were already primarily enriched in these elements and reached no regional extent.

### Metallogenetic Considerations: Implications for Exploration

A preliminary metallogenic classification of the rare metal mineralization in the Abu Rusheid-Sikait mineralized granites, including (a) the host petrogenesis, (b) the geodynamic setting, and (c) the major ore metal concentrating process, is:

- Post-tectonic mineralized granitoids and pegmatites, which are silica-rich (SiO_2_ = 69.7–82.7 wt%) and exhibit features of highly fractionated, alkaline to calc-alkaline, slightly peraluminous granites (A/CNK = 1.05–1.5) with progressive rare metal enrichment from biotite granites (ΣREEs: 92 ppm) through garnet-muscovite granites (ΣREEs: 1007 ppm) to pegmatites (ΣREEs: 1310 ppm) (Figs. 13, 14 and 15; Supplementary 6).
- Mantle and crustal melting by postcollisional lithospheric delamination and asthenospheric upflow.
- Mineralization is mainly related to fractionation processes (i.e., strong fractionation) and minor hydrothermal alteration, the latter leading to limited and local remobilization of rare metals (i.e., U, Pb associated with F and Fe) (Figs. 2, 3, 6, 8, 9, 10 and 11; Supplementary 6).

#### An Integrated Exploration Model and Newly Identified Prospective Zones

A primary outcome of this study is the development of an integrated exploration model (Fig. 16a) that successfully identifies new, high-potential zones for rare metal mineralization within the Abu Rusheid-Sikait area validated by whole rock geochemical analysis (Fig. 16b; Supplementary 6). By combining machine learning lithological classification (Fig. 5), alteration mapping (Fig. 6), radar-derived structural lineaments (Fig. 8), ground-truthing verification comprised 14 ASD field spectroscopy measurements (Fig. 7; Supplementary 3 − 2), 70 petrographic thin sections (Figs. 10 and 11), SEM/EDS (Fig. 12), geochemistry (Figs. 13, 14 and 15; Supplementary 5–7), we have generated a prospectivity map that highlights areas where the key ingredients for mineralization converge (Fig. 16a). While the initial enrichment of rare metals is magmatic, our findings demonstrate that hydrothermal overprinting, controlled by the structural framework, is critical for concentrating these elements into potentially economic deposits (Figs. 6, 8, 10 and 11; Supplementary 6). This integrated approach has revealed several previously undocumented targets that warrant detailed follow-up exploration. Based on the synthesis of our datasets, the following three high-priority zones are identified (arranged in order of prospectivity):

## High-Priority Zone 1: The Central Mineralized Core (GMGr-LMGr)

This zone, located centrally between Wadi Abu Rusheid and Wadi Sikait, represents the most promising exploration target in the study area. It is hosted within the most highly fractionated granitic units: the garnet-muscovite granites (GMGr), zinnwaldite-muscovite granites (LMGr), and associated pegmatites (Figs. 1 and 16a). The host rocks are geochemically confirmed to be the most enriched in primary rare metals (∑REEs up to 1007 ppm, Zr up to 3800 ppm, Nb up to 1370 ppm, Y up to 537 ppm, Ta up to 216 ppm, Th up to 503 ppm, and U up to 148 ppm; Supplementary 6). Moreover, the associated pegmatites have a high concentration of strategic metals (∑REEs up to 1310 ppm, Zr up to 4476 ppm, Nb up to 1500 ppm, Y up to 1436 ppm, Ta up to 145 ppm, Th up to 480 ppm, and U up to 411 ppm; Supplementary 6). Additionally, it exhibits the most widespread and intense alteration signatures, with overlapping phyllic (blue), muscovite (pink), and ferrugination (brown) alteration (Figs. 6a, b and h and 16a). As confirmed, ferrugination is directly correlated with secondary uranium enrichment (Figs. 2d and h, 5a, b and h, 10 and 11; Supplementary 3, 6, 7). This zone is situated in an area of high to moderate lineament density and is bounded by major NW-SE trending faults associated with the Najd fault system, which acted as primary conduits for hydrothermal fluids(Figs. 8 and 16a). This convergence of a fertile source rock, intense fluid-rock interaction, and structural permeability makes Zone 1 a prime target for structurally controlled REE, Zr, Y, U, Th, Nb, and Ta mineralization.

## High-Priority Zone 2: The Umm El-Kheran Pegmatite System (LPGr)

Located in the southeastern portion of the mapped area, Zone 2 is centered on the Li-rich pegmatite granites (LPGr) of the Umm El-Kheran pluton and south of zinnwaldite-muscovite granites (Figs. 1 and 16a). These pegmatitic granites represent the final, most volatile-rich stage of magmatic differentiation and are known hosts for lithium (in zinnwaldite) and other rare metals (Fig. 9n, o). The zone is clearly delineated by strong phyllic and muscovite alteration signatures, indicating significant post-magmatic fluid activity that could remobilize and concentrate elements (Figs. 6a and b and 16a). The area is cross-cut by a dense network of NE-SW and N-S trending faults that likely controlled the emplacement of the pegmatite bodies and subsequent hydrothermal fluids (Figs. 8 and 16a). This zone is highly prospective for Li, Nb, Ta, and Sn, and represents a classic target for rare-metal pegmatite granites exploration.

## High-Priority Zone 3: The Gabal Zabara Contact Zone

This prospective zone is located along the major thrust fault marking the northern contact between the ophiolitic mélange (Oph), gneisses (Gn), biotite granites (BGr), and the alkali feldspar granites (AFG) of Gabal Zabara (Figs. 1 and 16a). Its potential is derived from the regional-scale thrust fault, which represents a major crustal discontinuity, making it a highly favorable pathway for deep-seated hydrothermal fluids. The zone is characterized by extensive phyllic, argillic, and ferrous silicate alteration, indicating large-scale fluid flow along this structural corridor (Figs. 6a, c and d and 16a). Although the adjacent granites are less fractionated, the sheer scale of the fluid system suggests that it could have transported and deposited metals leached from a deeper, unexposed source.

In summary, this study has successfully integrated a multidisciplinary dataset to identify high-priority exploration targets, especially Zone 1, for rare-metal deposits in the Abu Rusheid-Sikait area, which is a validated methodology readily applicable to over 18 known rare-metal granite localities throughout the Egyptian Eastern Desert (Supplementary 1), and to similar post-collisional A-type granites across the Arabian–Nubian Shield.

### Data limitations and future works

While this study integrates automatic lineament extraction with field observations, geochemical data, and petrographic analysis, several limitations warrant acknowledgment:

1. Radar sensitivity: radar-derived lineaments represent surficial expressions and may not capture subsurface fault architectures; correlation with field-measured fault/fracture attitudes is essential for ground-truthing.
2. Automatic extraction limitations: lineament extraction algorithms are sensitive to topography and vegetation; in this study, we prioritize trends consistently identified across both ALOS PALSAR and Sentinel-1 datasets (NW-SE and NE-SW) and validate against field observations.
3. Spatial resolution: the 30 m pixel resolution of PRISMA and radar imagery limits direct fracture mapping; lineament density maps represent an aggregate proxy for fault density rather than complete structural enumeration.
4. Future work will involve targeted field investigations, including systematic kinematic measurements of fault and fracture attitudes (strike, dip, and lineation), to enable direct quantitative comparison with radar-derived lineament orientations. Fluid-inclusion studies will be conducted to constrain the physicochemical conditions (temperature, pressure, and fluid composition) associated with hydrothermal mineralization. In addition, integrated geophysical investigations (e.g., radiometric, magnetic, and electrical methods) are required to delineate the subsurface continuity of surface structures and to identify and characterize zones of radioactive enrichment.

## Conclusions

This integrated study of the Abu Rusheid-Sikait area provides a comprehensive model for rare metal mineralization in post-collisional A-type granites within the Arabian-Nubian Shield. The primary conclusions are:

1. The application of machine learning algorithms (SVM and RF) to hyperspectral PRISMA data provides an accurate and efficient method for lithological discrimination and mapping of hydrothermal alteration zones (phyllic, argillic, propylitic, and ferrugination) in complex granitic terrains.
2. The enrichment of rare metals is the result of a two-stage process. First, extreme magmatic fractionation of a peraluminous, A-type granitic magma led to the primary concentration of REEs, Zr, Nb, and Th in accessory minerals like zircon, columbite, and monazite. Second, post-magmatic hydrothermal fluids, channeled along major structural lineaments related to the Najd fault system, overprinted these primary assemblages, leading to localized remobilization and secondary enrichment of elements like U and Pb.
3. The distribution of mineralization is strongly controlled by the intersection of highly fractionated granitic units with regional fault and shear zones. Hydrothermal alteration, particularly ferrugination, played a significant role in the secondary concentration of uranium.
4. The investigated rocks, particularly the muscovite (-garnet-)(-zinnwaldite)-rich granites and pegmatites, should be considered as a potential resource to warrant exploration for REEs and other rare metals. The integrated exploration model developed in this study has successfully identified three high-priority zones that warrant further investigation. This methodology serves as a guide for future exploration efforts in similar geological settings across the Eastern Desert of Egypt and the wider ANS.

## Supplementary Information

Below is the link to the electronic supplementary material.

Supplementary Material 1

## Data Availability

All the data derived from this research are presented in the enclosed figures and supplementary materials.
